# Sustainable and Integral Valorization of *Dosidicus gigas* Pen Waste: Combined Production of Chitosan with Antibacterial Properties and Human and Marine Probiotics

**DOI:** 10.3390/md23100382

**Published:** 2025-09-27

**Authors:** Marta Lima, Adrián Pedreira, Noelia Sanz, José Antonio Vázquez, Míriam R. García, Filipe Mergulhão, Jesus Valcarcel

**Affiliations:** 1 LEPABE, ALiCE, Faculty of Engineering, University of Porto, Rua Dr. Roberto Frias, 4200-465 Porto, Portugal; up201604683@edu.fe.up.pt (M.L.); filipem@fe.up.pt (F.M.); 2 Recycling and Valorization of Waste Materials (REVAL), Marine Research Institute (IIM-CSIC), Eduardo Cabello 6, 36208 Vigo, Spain; apedreira@iim.csic.es (A.P.); nsanz@iim.csic.es (N.S.); jvazquez@iim.csic.es (J.A.V.); 3 Bioprocess Engineering (Bio2eng), Marine Research Institute (IIM-CSIC), Eduardo Cabello 6, 36208 Vigo, Spain; miriamr@iim.csic.es; 4 Scientific Technical Support Unit (UACT), Marine Research Institute (IIM-CSIC), Eduardo Cabello 6, 36208 Vigo, Spain

**Keywords:** chitin, chitosan, *Dosidicus gigas* pen, by-product and effluent valorization, antimicrobial activity, probiotic production

## Abstract

This study details a biorefinery approach to valorize *Dosidicus gigas* squid pen waste. The process starts with the enzymatic deproteinization of squid pens, which prove effective with both Alcalase and Novozym, with the latter exhibiting a slightly higher efficiency to yield a material with 73% chitin content. Subsequent alkaline hydrolysis produces highly deacetylated chitosan (>90% degree of deacetylation), followed by controlled depolymerization to obtain polymers with molecular weights ranging from 50 to 251 kDa. Both native and depolymerized chitosan exhibit antimicrobial activity against *Escherichia coli* and *Bacillus cereus*, with *B. cereus* demonstrating greater resistance to chitosan compared to *E. coli*. The research also explores the bioconversion of deproteinization and deacetylation effluents. Deproteinization effluents prove superior in sustaining microbial growth, supporting comparable growth and lactic acid production for human probiotic strains (*Lactobacillus plantarum* and *Leuconostoc mesenteroides*) when substituting commercial peptones. Marine bacteria (*Pseudomonas fluorescens* and *Phaeobacter* sp.) show lower productivity. Integrating these processes into a biorefinery framework enables the conversion of 1 kg of dry squid pens into 350 g of chitosan, and facilitates the production of 937–949 g of lactic acid using human lactic acid bacteria cultures in media formulated with squid pen-derived effluents, glucose, yeast extract, and mineral salts. This integrated approach highlights the potential for maximizing resource utilization from squid pen waste, reducing environmental impact and generating high-value bioproducts.

## 1. Introduction

*D. gigas* is the largest and most abundant oceanic cephalopod marine invertebrate in the coastal waters of the Eastern Pacific Ocean [1,2]. Due to its rapid growth and expanding distribution, *D. gigas* was the most heavily fished squid species between 2018 and 2021. In 2021, over 1 million tonnes of *D. gigas* were caught, accounting for 16.2% of the total molluscs captured worldwide [3,4,5,6], followed by *Illex argentinus* (7.2%) and *Mizuhopecten yessoensis* (5.9%). Considering an average edible flesh yield of 70% [7] the annual waste generated from *D. gigas* processing is estimated at 300 thousand tonnes, contributing to disposal and environmental challenges [8]. Addressing this issue from a circular economy perspective could involve repurposing squid pen by-products for valuable material production. Through a shell biorefinery approach, squid by-products can be sustainably converted into value-added products, such as chitin and its derivatives, benefiting both the environment and the economy [9].

Chitin is a long-chain natural polysaccharide with N-acetylglucosamine units linked by β-(1→4) glycosidic bonds. As the second most abundant natural polysaccharide after cellulose, it can be extracted from the cell walls of microorganisms, as well as from the exoskeletons of crustaceans, mollusks, insects, and algae [10,11,12]. Chitin exists in three forms—α-, β-, and γ-chitin—depending on the original source, each with distinct physicochemical properties. Chitin derived from crustacean shells (α-chitin) has a tightly packed structure due to strong intermolecular hydrogen bonds between the microfibrils organized in parallel. In contrast, β-chitin, typically sourced from squid pens, features antiparallel chains with weaker intra- and intermolecular forces, resulting in higher solubility, water-absorbing capacity, and reactivity in alkali solution [13], making it more suitable for various applications.

Traditional methods for extracting chitin from marine by-products involve the use of hazardous chemicals and are energy-intensive. The extraction process from crustacean shells usually includes three steps: demineralization with hydrochloric acid; deproteination using hot alkali; and bleaching of carotenoid pigments with oxidizing agents [12,14]. Squid pens, however, contain lower amounts of minerals and carotenoids, making demineralization and decoloration treatments unnecessary, which presents an advantage in terms of sustainability [15]. Although squid pen processing only requires deproteination, the use of strong alkali compounds affects chitin depolymerization and requires effluent neutralization. To overcome these limitations, sustainable and eco-friendly extraction methods, including fermentative and enzymatic strategies, have been developed. Lactic acid bacteria and proteases have been successfully used to extract chitin from crustacean shells, though their application to squid pens remains limited [16].

Chitin’s poor solubility and low reactivity often constrain its usage, prompting the production of improved deacetylated derivatives through chemical hydrolysis in alkaline environments. In this process, N-acetylglucosamine units of chitin are partially deacetylated to glucosamine units, yielding chitosan—a copolymer of β-(1→4)-linked N-acetyl-*D*-glucosamine and *D*-glucosamine units. Chitosan’s increased solubility in dilute acids, along with the amenability of its reactive functional groups to chemical modification, enhances its biocompatibility and antimicrobial properties, broadening its applications [17]. Chitosan, either in its native form or chemically modified, has attracted significant interest for several applications, including food and pharmaceutical industries, biomedicine, agriculture, water treatment, and cosmetics [18]. This is due to its unique biological properties, including non-toxicity, biocompatibility, biodegradability, good film-forming properties, chemical stability, non-allergenic behavior, low cost, and wide natural availability [19]. Furthermore, chitosan exhibits intrinsic antimicrobial activity against both Gram-positive and -negative bacteria, filamentous fungi, and yeasts [20], making it an appealing polymer for the development of antimicrobial coatings. Its antimicrobial efficacy is influenced by several environmental parameters, including pH, surrounding components and type of microorganisms, and chitosan structural properties, such as the source, derivative form, concentration, degree of deacetylation, and molecular weight [21].

The effluents generated during the enzymatic deproteination of squid pen, and to a lesser extent those derived from the alkaline deacetylation of chitin, contain nitrogen-rich compounds resulting from protein degradation. Valorizing these effluents could significantly enhance the sustainability of chitin and chitosan production. However, research on this topic remains scarce. To date, only one study has explored this potential, wherein the proteinaceous material present in the alkaline deproteination solution of arrow squid pen was hydrolyzed to obtain antioxidant and antihypertensive peptides [22]. An alternative to protein hydrolysis involves the incorporation of these effluents into culture media as nitrogen sources for biotechnological applications. This approach has proven viable for other fish-processing effluents, such as gelatin production from fish skin or fish canning wastewaters, supporting the growth of various microorganisms capable of producing metabolites of interest, including lactic and fatty acids [23,24,25,26].

The present study aims to: (i) optimize the conditions for extracting chitin from *Dosidicus gigas* squid pen by-products using enzymatic treatments; (ii) depolymerize native chitosan to obtain derivatives of varying molecular weights; (iii) sustainably recycle the generated protein and alkaline effluents for use in other biotechnological processes; (iv) evaluate the antimicrobial activity of *D. gigas* chitosan against *Escherichia coli* and *Bacillus cereus*. To the best of our knowledge, this is the first study to optimize chitin extraction from *D. gigas* pens using enzymatic treatments.

## 2. Results and Discussion

### 2.1. Pen Deproteinization for Chitin Production

The basic composition of fresh squid pens remains stable and independent of the season of squid capturing [27]. The average proximate composition values (%, *w*/*w*) are as follows: moisture, 63.3 ± 1.6%; organic matter, 1.1 ± 0.2%; and ashes, 35.6 ± 1.3%. After drying at 80 °C overnight, the chemical composition (%, *w*/*w*) is 2.2 ± 0.5% of ashes, 1.2 ± 0.3% of total lipids, 52.7 ± 4.9% of protein (expressed as the total sum of amino acids), and 43.9 ± 2.2% of chitin. The total nitrogen content is 8.5 ± 0.4%. Regarding heavy metals, dried samples contain 0.022 ± 0.007 ppm of Hg, 0.066 ± 0.011 ppm of Pb, and 0.292 ± 0.028 ppm of Cd.

**Figure 1:**
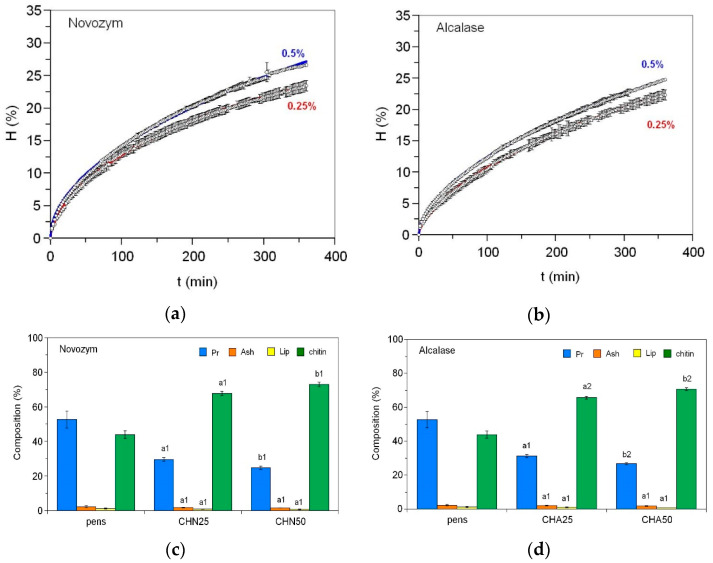
Dynamics of the hydrolysis degree of squid pens using Novozym (**a**) and Alcalase (**b**) at 0.25% and 0.5% concentrations and chemical composition of the initial pens and the chitins obtained after 6 h of hydrolysis with Novozym (**c**) and Alcalase (**d**) using 0.25% (CHN25 and CHA25) and 0.5% (CHN50 and CHA50) of the respective proteases. Experimental data were fitted to the Weibull equation [16]. Different letters (a, b) indicate significant differences (*p* < 0.05) between protease concentrations within the same enzyme treatment (CHN25 vs. CHN50, and CHA25 vs. CHA50). Different numbers (1, 2) denote significant differences (*p* < 0.05) between enzymatic hydrolysis at the same concentration (CHN25 vs. CHA25, and CHN50 vs. CHA50).

We tested two commercial endoproteases for the deproteinization of squid endoskeletons at two concentrations (0.25 and 0.5%, *v*/*w*) over a 6 h proteolysis period, following the conditions previously optimized for *Illex argentinus* pens [16]. In all kinetics studied, the dynamics of the hydrolysis degree (H, in %) show hyperbolic profiles easily modeled by time-dependent Weibull equation [16] (Figure 1, top). The highest enzyme concentrations result in the highest final hydrolysis degrees: 26.6% and 24.8% for CHN50 and CHA50, respectively, compared to 23.4% and 22.4% for 0.25% of Novozym and Alcalase. The composition of the obtained products in dry weight (%, *w*/*w*) is summarized in Figure 1 (bottom). The total protein content in chitin samples decreased from 52.7% (in dry pens) to 29.6% with 0.25% Novozym and to 24.8% at the higher concentration of this protease. Alcalase treatment leads to slightly lower protein removal, with residual protein contents of 31.3% and 26.7% for the lower and higher enzyme concentrations, respectively. The differences between enzyme concentrations are statistically significant, as is the comparison between CHN50 and CHA50 (*p* < 0.05). In all cases, ash content remains below 2%, and total lipid content does not exceed 1%. The chitin content reaches a maximum of 73% in samples treated with 0.5% Novozym, followed by Alcalase at the same concentration. Novozym exhibits a small, but significant (*p* < 0.05), higher deproteinization efficiency, yielding samples from *D. gigas* pens slightly richer in chitin.

Compared to traditional alkaline deproteinization, enzymatic hydrolysis offers a more sustainable alternative. It avoids the use of strong alkali, which can lead to partial depolymerization of chitin and generate effluents requiring neutralization [13]. Enzymatic methods also preserve the native polymer structure and yield protein-rich effluents that can be valorized as microbial nutrients, improving overall process economy [15].

### 2.2. Chitin Deacetylation for Chitosan Production

Chitin samples obtained with each enzyme were pooled, i.e., CHA25 and CHA50, and CHN25 and CHN50, since the differences in composition, while statistically significant, are not substantial in magnitude, as described in the previous section. To obtain chitosan, these chitin samples require harsh conditions to cleave the C-N bond in N-acetyl glucosamine and release the acetyl group. Chitin deacetylation protocols typically describe heterogeneous reactions in which solid chitin is treated with a strong alkaline solution at relatively high temperatures under agitation. The literature reports wide variation in processing conditions, with alkali concentration (usually NaOH or KOH) ranging from 30% to 50%, temperature from 80 to 120 °C, and reaction time spanning from minutes to days [26,28].

Harsher conditions result in a higher degree of deacetylation, which in turn generally correlates with enhanced antibacterial activity [29,30]. Therefore, in the present study, we chose relatively high alkali concentrations and extended reaction times. Specifically, we tested the effect of treating chitin deproteinized with Novozym and Alcalase under the following conditions: 12.5 M NaOH for 12 h (CSN1 and CSA1, respectively), 15 M NaOH for 6 h (CSN2 and CSA2, respectively), and 15 M for 24 h (CSN3 and CSA3, respectively), setting the temperature at 90 °C. In all cases, the deacetylation degree is high, exceeding 85%, with comparable values between chitin samples deproteinized with either Alcalase or Novozym (Table 1). Although the differences are minor, treatment with 15 M NaOH for 24 h yields the highest deacetylation degree (91.5%), alongside high purity: 96.7% of the material is chitosan, with only 3.1% of total protein, 0.2% of ash, and no detectable lipids. Based on these results, we selected this condition to upscale chitosan production for further testing. This concentration is within the range commonly used for high-degree deacetylation (>90%) [28]. Although a strong alkali is still required for deacetylation, the prior enzymatic deproteinization reduces residual protein content, minimizing the need for repeated alkali exposure and improving chitosan purity.

### 2.3. Chitosan Depolymerization

**Figure 2:**
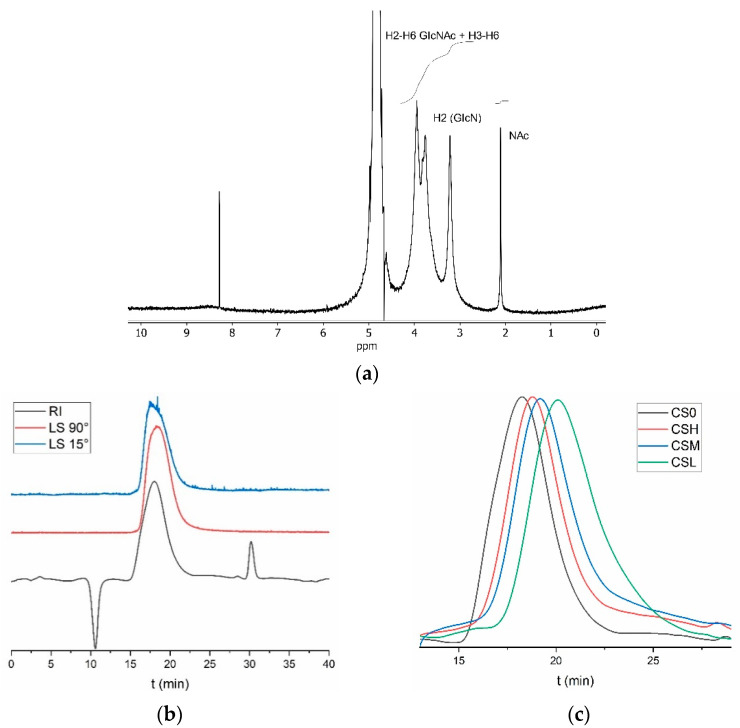
**^1^**H NMR spectrum of chitosan (CS0) in 0.056M d-TFA in D2O at 400 MHz. GlcN: glucosamine, GlcNAc: N-acetylglucosamine, and NAc: N-acetyl. Deacetylation degree 91.5% (**a**). GPC eluogram of CS0 (black line: refractive index detector; red line: right angle light scattering detector; blue line: low angle light scattering detector (**b**). GPC eluograms of chitosan depolymerization (refractive index detector) (**c**).

Chitosan production was upscaled to prepare enough quantity for subsequent depolymerization and antimicrobial activity tests. *D. gigas* pens were deproteinized using 0.5% Novozym at 60 °C and pH 8.5, with a solid:liquid ratio of 1:10, agitation at 200 rpm, and a reaction time of 6 h in a 5 L reactor. The resulting chitin was then deacetylated with 15 M NaOH at 90 °C for 24 h, yielding native chitosan (CS0). Characterization of CS0 by GPC and ^1^H NMR reveals an average number molecular weight (Mn) of 251 kDa and a deacetylation degree of 91.5%, respectively (Table 2, Figure 2a,b). ^1^H NMR also displays the typical signals in chitosan, the methyl protons of the N-acetyl group at 2.1 ppm, the H-2 proton of the D-glucosamine unit at 3.2 ppm, and the overlapping signals from the H-3, H-4, H-5, and H-6 protons of both the D-glucosamine and N-acetyl-D-glucosamine units from 3.4 to 4.4 ppm, confirming the identity of the polymer. The anomeric protons of both acetylated and deacetylated units are not visible because of overlap with the solvent residual peak. The reported Mw of *D. gigas* chitosan varies widely, partially due to differences in analytical techniques. Viscosity measurements tend to yield considerably higher molecular weight estimates compared to those obtained by gel permeation chromatography coupled with light scattering detection. In the first case, studies report values of 442–12,000 kDa [31,32], and in the second of 92–267 kDa [33,34,35], in line with the molecular weight determined in the present study.

The reaction of native chitosan (CS0; 251 kDa) with increasing amounts of nitrite results in a stepwise reduction in molecular weight, yielding high- (CSH; 137 kDa), medium- (CSM; 94 kDa), and low-molecular-weight (CSL; 50 kDa) chitosan (Table 2). We followed the depolymerization kinetics by injecting reaction aliquots onto the GPC system. Polymeric peaks elute at higher volumes (i.e., longer elution times) as the molecular weight decreases, so we injected aliquots at regular intervals until we observed steady elution volumes corresponding to the molecular weights selected. Figure 2c displays the final eluograms for native and depolymerized chitosan. These four chitosan materials were used for subsequent antimicrobial activity assays.

### 2.4. Antimicrobial Activity

**Figure 3:**
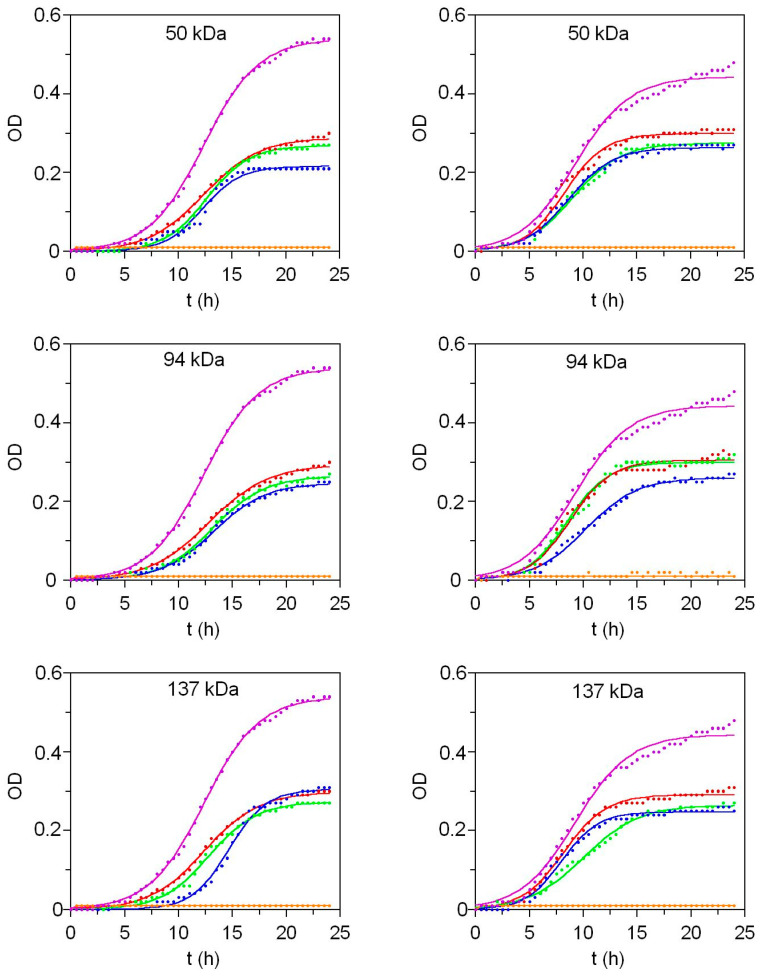

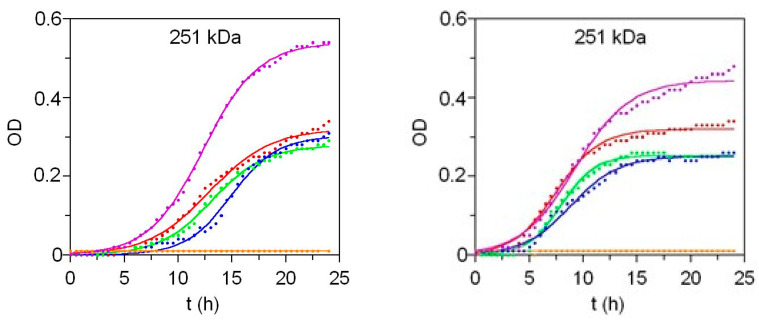
Growth dynamics of *E. coli* (**left**) and *B. cereus* (**right**) in the presence of different concentrations of chitosan with varying molecular weights: 0 mg/L (●), 3.9 mg/L (●), 7.8 mg/L (●), 15.6 mg/L (●), and 31.2 mg/L (●). Experimental data (points) were fitted to the logistic growth equation (solid lines).

The antimicrobial activity of β-chitosan with different molecular weights was evaluated against vegetative cells of *Escherichia coli* and *Bacillus cereus*, two common foodborne bacterial species, by monitoring the planktonic growth of these microorganisms in the presence of different β-chitosan concentrations. Bacterial growth kinetics were analyzed based on optical density (OD) at 600 nm (Figure 3). Experimental data were fitted to the logistic growth model [1] (Table S5), and the numerical values of the corresponding parameters are reported in Tables S7 and S8 (Supplementary Materials). In this study, OD was used as the dependent variable instead of biomass (X), as specified in the logistic equation from Table S5. The key parameters of this mathematical model include OD_m_ (maximum optical density), ν_OD_ (maximum optical density rate), and λ_OD_ (lag phase of the optical density).

Both *E. coli* and *B. cereus* show susceptibility to β-chitosan, as shown in Figure 3. The growth dynamics and the corresponding numerical parameters are influenced by the presence of the biopolymer, with effects varying between bacterial strains. Regarding *E. coli*, increasing chitosan concentration leads to a pronounced reduction in the maximum growth (OD_m_) and a significant extension of the lag phase (λ_OD_) across all molecular weight variants of chitosan (Table S7). Conversely, in *B. cereus*, while OD_m_ is significantly reduced (*p* < 0.05), the prolongation of the lag phase is not statistically significant (*p* > 0.05) (Table S8). In both cases, the highest maximum growth rates occur in control cultures without chitosan.

Despite these differences, both bacterial strains share a Minimum Inhibitory Concentration (MIC) of 31.2 mg/L for chitosan regardless of molecular weight, as this is the concentration at which no growth is detected. The growth curves at sub-MICs further indicate that the antimicrobial activity of chitosan is concentration-dependent, although the differences observed are relatively small. However, a pronounced shift occurs between the MIC (31.2 mg/L) and the MIC/2 (15.6 mg/L), where bacterial growth transitions from complete inhibition to only a 50–60% reduction. This finding raises concerns about the conventional use of twofold concentration increments in MIC determinations and growth inhibition assays for non-antibiotic antimicrobial compounds, such as food disinfectants or preservatives [36].

On the other hand, modelling of inhibition-concentration (IC) profiles in terms of OD_m_, allows the establishment of dose–response relationships between the concentration of chitosan and growth inhibition (Figure S2, Supplementary Materials). IC profiles are reasonably predicted by the Weibull equation, with IC_50_ values confirming the higher resistance of *B. cereus* to chitosan compared to *E. coli* (Table S9, Supplementary Materials). Regarding chitosan molecular weight, *E. coli* exhibits a dependency on chitosan size, with the lowest IC_50_ observed for the smallest chitosan derivative. In contrast, this behaviour is not evident for *B. cereus*.

The finding that, based on IC_50_, the Gram-positive *B. cereus* exhibits higher resistance to chitosan than the Gram-negative *E. coli* contradicts the Spaulding classification, which traditionally assumes Gram-negative bacteria to be more resistant to biocidal compounds than their Gram-positive counterparts. Yet, recent evidence suggests that this assumption may not always hold true, as several studies have revealed unexpected resistance profiles that challenge the established dogma related to the resistance scale [37]. The specific case of chitosan is no exception. Studies in the literature report three possible scenarios: *B. cereus* appearing more resistant than *E. coli* [38], both species showing similar resistance [20,38], or *B. cereus* being more susceptible [39]. Importantly, these comparisons are almost exclusively based on MIC values, which in the present study demonstrate an equal susceptibility for *E. coli* and *B. cereus*, rather than on IC_50_ determinations, which indicate here that *E. coli* is actually more sensitive than *B. cereus*. In any case, the reduced susceptibility (according to IC_50_) of *B. cereus* compared to *E. coli* when exposed to chitosan found in the present study can be explained based on the mechanism of action of chitosan, its specific attributes and the target species. The primary hypothesis explaining the antimicrobial action of chitosan is fundamentally attributed to the electrostatic interaction between its protonated amino groups and the anionic components on the microbial cell surface. This interaction seems to lead to membrane disruption and cell leakage [36,40]. Nevertheless, the effectiveness of this interaction is heavily influenced by a number of factors, such as molecular weight and degree of deacetylation, as well as by the distinct structural characteristics of the cell envelopes [41].

The outer membrane (OM) of Gram-positive bacteria has a significantly thicker peptidoglycan wall (30 to 40 layers) compared to Gram-negative. This peptidoglycan layer contains anionic components such as lipoteichoic acids and wall teichoic acids, which attract the cationic chitosan [39,41]. However, the substantial thickness of the peptidoglycan wall also acts as a robust physical barrier, which may impede high-molecular-weight (HMW) chitosan molecules employed in the present study (50–251 kDa) from reaching the cytoplasmic membrane. In contrast, the OM of Gram-negative bacteria is stabilized by divalent cations, with highly anionic lipopolysaccharides providing numerous negatively charged sites [42]. Chitosan acts by competing with stabilizing divalent cations, leading to the destabilization and rupture of the OM barrier [40]. The greater susceptibility of *E. coli* observed in this study can be explained by the efficacy of the highly charged chitosan, which effectively disrupts the OM through electrostatic displacement of metal ions, thus overcoming the barrier function [41]. In contrast, the rigid and thick peptidoglycan layer of *B. cereus* offers increased structural protection against the HMW chitosan molecules [42].

HMW chitosan generally exerts its antimicrobial effect through extracellular mechanisms, such as forming an impermeable layer on the cell surface that blocks nutrient transport and chelates essential metallic ions [36,40,42]. Low-molecular-weight (LMW) chitosan (5–10 kDa) is believed to penetrate the cell wall and membrane to interfere with intra-cellular processes like DNA/RNA synthesis [42]. A prior investigation involving pronase-depolymerized LMW chitosan (8.5–9.5 kDa) found that it achieved complete lysis more efficiently than native chitosan (∼71 kDa) for both *B. cereus* and *E. coli* [39]. That same study reported greater inhibitory activity toward *B. cereus* than *E. coli* at low concentration, contradicting the results presented here employing HMW chitosan. The fact that the present study utilizes significantly larger molecules might explain the reduced efficiency against *B. cereus*, as penetrating its thick peptidoglycan layer is more challenging for larger polymers such as HMW chitosan [40,41]. Furthermore, while *E. coli* shows a modest dependence on chitosan molecular weight (the 50 kDa derivative was slightly more effective), *B. cereus* exhibits no such dependency within the tested HMW range. This suggests that the thick cell wall structure of *B. cereus* minimizes the role of molecular size until the molecular weight is drastically lowered (below the range tested here). Finally, the chitosan tested in the present study possess a high deacetylation degree (>90%). This level of deacetylation ensures a high density of protonated amino groups at the pH of the assay (6.2), which maximizes the cationic charge of chitosan. Highly deacetylated chitosan generally exhibits stronger antimicrobial activity due to enhanced electrostatic interactions with the microbial cell surface [36]. The high deacetylation degree chitosan used here would be expected to maximize binding to the surface of both bacterial strains. However, this interaction might have been stronger in the case of *E. coli* due to the highly anionic lipopolysaccharides found in the OM [36,40], thus leading to a proportional greater damage in comparison with the resistance provided by the thick peptidoglycan wall of *B. cereus*.

### 2.5. Bioconversion of Residual Effluents

#### 2.5.1. Production of Human Probiotics

**Figure 4:**
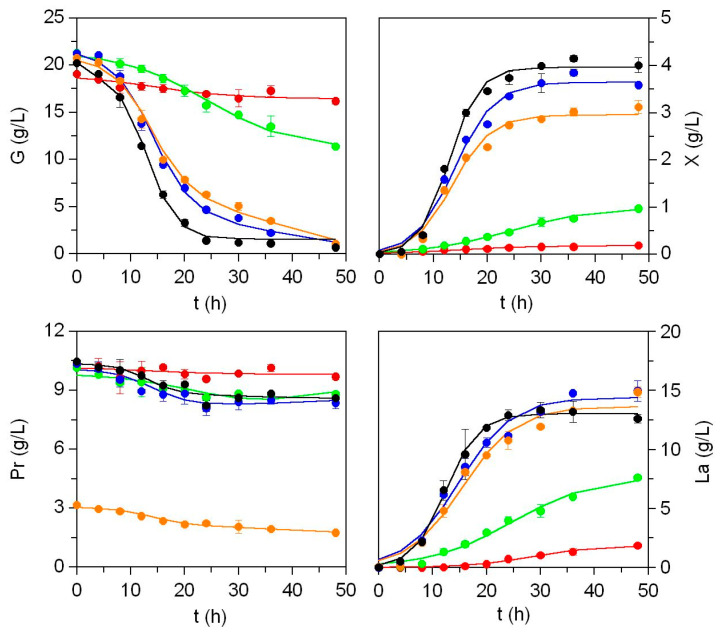
Biomass dynamics, lactic acid production, and nutrient uptake for *Leuconostoc mesenteroides* CECT 4046 (Ln) growing in the formulations described in Table S3. Experimental data for biomass (*X*), lactic acid (*La*), proteins (*Pr*) and glucose (*G*) were fitted to the equations displayed in Table S5 (solid lines). Growth media are represented as follows: Medium A (●), medium B (●), Medium C (●), Medium D (●) and MRS (●). Error bars represent the confidence intervals for *n* = 3 and α = 0.05.

Figure 4 and Figure S3 (Supplementary Materials) show the experimental results of the Lb and Ln cultures, highlighting significant differences in bacterial growth dynamics and lactic acid production across the different media evaluated, including the MRS control. Acetic acid data were excluded, as its production did not exceed 1.5 g/L in any condition. Glucose and protein consumption follow similar patterns in both lactobacteria strains, with inverse profiles to biomass accumulation and lactic acid production, characterized by decreasing kinetics. The highest substrate uptakes, leading to complete glucose depletion, occur in nutrient-rich media (C, D and MRS), together with a poor assimilation capacity of medium A and a slower or delayed nutritional ability of medium B. The amino acid composition of both effluents (Table S1, Supplementary Materials) is rich in essential amino acids (>39%), and none of the amino acids (except hydroxyproline) are absent from such substrates.

From a statistical perspective, the unstructured model summarized in Tables S5 and S6 (Supplementary Materials) demonstrates to be an excellent mathematical tool for predicting the fermentation dynamics of Lb and Ln (Tables S10 and S11, Supplementary Materials). Robustness of the model is supported by the following observations: 1) all kinetic equations are statistically significant (*p*-values < 0.05); 2) approximately 85–90% of the parameters are statistically meaningful; and 3) the coefficient of determination (R_2_) exceeds 0.962 in most cases, except for glucose and protein consumption in medium A.

For both bacterial species (Tables S10 and S11, Supplementary Materials), alternative media formulated from effluents simulating MRS composition without commercial peptones (media C and D) support maximum biomass yields (*X_m_*) comparable to those observed in control cultures executed in MRS (ChE effluent was slightly less nutritious). Medium A, by contrast, does not support remarkable growth (*X_m_* < 0.2 g/L). In medium B, despite a prolonged lag phase (*λ_x_* = 11 h), the biomass concentrations reached are reasonable (0.7 g/L and 1 g/L in Lb and Ln, respectively), considering its cost-effective composition: glucose + CE + mineral salts. For the remaining parameters, there are no significant differences among cultures grown in media C, D and MRS (*p* > 0.05), with comparable maximum biomass production rates and lag phases. Compared to the minimal media (A and B), the most productive media (C and D) facilitates more rapid growth, exhibiting significantly shorter lag phases.

Regarding the analysis of biomass yields (Tables S6, S10 and S11, Supplementary Materials), the results highlight significant differences in metabolic efficiency among the tested media. Biomass production as a function of glucose consumption (*Y_x_*_/*g*_) is notably higher in media C, D and MRS, with values ranging from 0.146 to 0.164 g G/g X for Lb and 0.218 to 0.222 g G/g X for Ln. Similar responses when evaluating biomass formation in relation to protein consumption (*Y_x_*_/*P*_), also exhibit the highest values recorded in media D, C and MRS for Lb, while for Ln the ranking is D > MRS > C. In both parameters, the fermentation process with Ln demonstrates greater productivity and efficiency in biomass formation compared to Lb. The terms related to cell maintenance based on glucose and protein (*m_g_* and *m_p_*) are mostly not statistically significant (Student’s *t* test). However, for *m_g_*, the values observed in media D and C, in that order, exceed those calculated for MRS. Although *m_g_* is not a strictly defined parameter, its interpretation is linked to the regulation of cellular functionality, differentiation, nutritional needs, and the viability of the genetic and metabolic resources of the bacterial population [43].

In parallel with biomass production, the values of maximum lactic acid production (*L_m_*) and the maximum production rates of lactic acid (*ν_L_*) indicate the ability of media C and D to sustain lactic acid synthesis. These results are statistically comparable to those obtained with MRS (*p* > 0.05), further demonstrating the suitability of these alternative media. In contrast, the maximum production of lactic acid in medium A is negligible (<1.9 g/L), whereas medium B supports significant production (around 8 g/L in both LAB strains). All media, except A, display the highest lactic acid production efficiencies relative to biomass (*Y_L_*_/*x*_). However, when considering lactic acid yield relative to glucose consumption (*Y_L_*_/*g*_), the following distinct pattern emerges: media A and B exhibit significantly higher (*p* < 0.05) efficiency compared to the other formulations.

Altogether, these findings highlight the potential of residual squid pen effluents as a valuable source of assimilable peptones, compared to those included in MRS (meat extract and bactopeptone), effectively supporting both the growth of two lactobacteria and the production of lactic acid. These results align with previous studies on peptones recovered from cephalopods [44], salmon by-products [45], and cod [46], as well as effluents derived from the isolation of marine gelatin [25]. Notably, the observed performance surpasses that reported for peptones derived from salmon by-products fermented by *L. casei* [47], further supporting the feasibility of using marine-derived peptones in biotechnological applications.

#### 2.5.2. Production of Marine Probiotics

Figure S4 and Table S12 (Supplementary Materials) display the experimental data for Pha and Pf cultures, conducted in media A–D and MM (Table S4, Supplementary Materials), along with the simulations based on Equations [1] and [4] (detailed in Table S5 of Supplementary Materials). Cultures in medium B did not lead to observable growth for either bacterial strain, and therefore, these results are not included in the figures. A clear distinction emerges between the performance of MM and the alternative media, particularly in terms of protein consumption efficiency, which is notably higher in media C and D. Medium A demonstrates remarkable efficiency by supporting the production of a substantial biomass yield with minimal protein consumption.

The maximum biomass production achieved in the three media containing residual effluents is significantly lower than that obtained in MM, with medium C being the most effective among the alternative formulations. Regarding the *ν_X_* values in Pf, the highest maximum biomass production rates are obtained in medium C, though differences are not statistically significant among the four tested formulations (*p* > 0.05). Similarly, lag phase durations across the culture media show no significance. In contrast, for Pha, MM leads to *X_m_* and *ν_x_* values approximately twice those achieved in media A, C, and D.

Regarding productive yield for Pf, MM shows the highest values (*Y_x_*_/*P*_ = 1.48 g *X*/*g* Pr), followed by medium D (*Y_x_*_/*P*_ = 0.74 g X/g Pr). The biomass productive capacity in relation to the soluble protein consumption is considerably lower in media A and C (0.25 g X/g Pr and 0.48 g X/g Pr, respectively). In line with this behavior, the corresponding Pha yields in A and C are also lower than MM and D, which, in this case, is the most productive. The cell maintenance term (*m_p_*) exhibited the highest values for both marine bacteria in medium D, further underscoring its role in sustaining bacterial viability under these conditions.

In line with the findings related to LAB, CE and ChE emerge as an interesting source of protein material (high presence of essential amino acids), useful as an ingredient for low-cost alternative media formulated for the cultivation of probiotic marine bacteria. It must be considered that the new marine medium proposed in this work (medium C) simply consists of filtered seawater, 1 g/L of yeast extract, and CE. Therefore, while the amino acid profile of the effluents is very complete, it is possible that the specific type, size, or structure of the proteins within these effluents may not be fully optimized to replace the neopeptone used in the commercial medium (MM).

**Figure 5:**
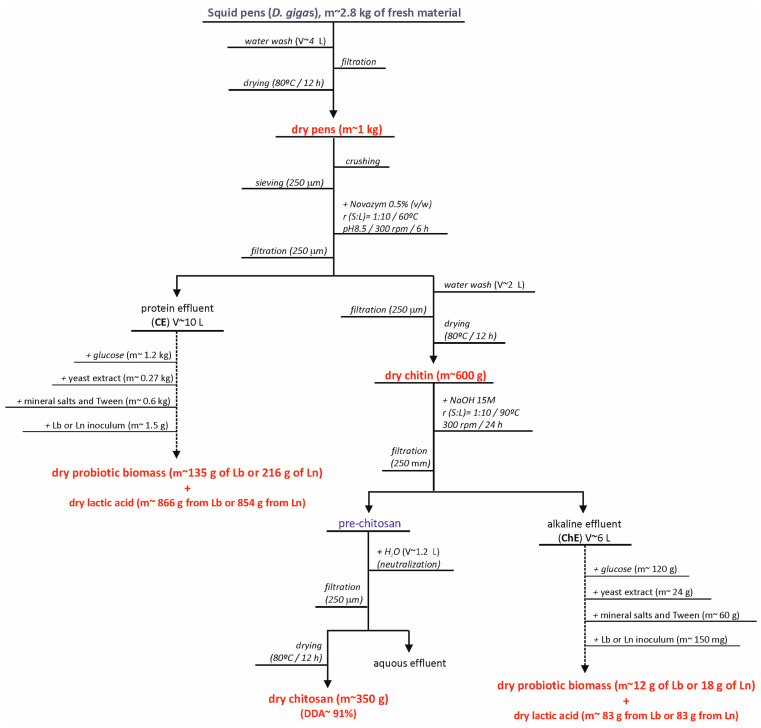
Flowchart of chitosan balance mass produced on optimal conditions, including the production of human probiotics using the residual effluents generated in the deproteinization and deacetylation stages, for 1 kg of initial by-product (pens of *Dosidicus gigas*).

Finally, to assess the overall potential of these effluents for integrated biorefinery applications, we calculated the complete mass balance, as shown in Figure 5 and Table S15. This biorefinery approach was specifically developed for the production of human probiotics using residual effluents CE and ChE. Results indicate that for every kg of initial squid pens, it is possible to produce 350 g of chitosan (DDA = 91%), 147 g of *L. plantarum* (or 234 g of *L. mesenteroides*), and from 937 to 949 g of lactic acid. The latter bioproduction achieved the highest value on complete medium formulated with glucose, chitin effluents, yeast extract and salts simulating MRS. In culture broths formulated with glucose + chitin effluents, glucose + salts + chitin effluents, glucose + salts + yeast extract and glucose + salts, the production of lactic acid was only 12%, 53%, 48% and 20%, respectively, of that obtained in the mentioned complete medium. Notably, washing waters and aqueous effluents are excluded from these calculations due to their low concentrations of proteins and total organic matter when compared to the primary effluents under study. These outcomes align with previous research exploring the integrated valorization of fish skin wastes, where oil, gelatin, gelatin hydrolysates, lactic acid and lactic acid bacteria biomass were successfully produced [25].

## 3. Materials and Methods

### 3.1. Squid Pen By-Products

Frozen squid pens from *Dosidicus gigas* were kindly provided by Nueva Pescanova S.A. (Redondela, Pontevedra, Spain). These by-products were generated during the evisceration of jumbo squid captured on the Pacific coast by Nueva Pescanova’s fleets. The specimens were shipped frozen from the company’s facilities in Peru. Upon thawing, the pens were thoroughly washed with water, oven-dried overnight at 80 °C, and subsequently ground through 250 and 500 µm meshes. Fresh and dried squid pens were characterized regarding their water and ash content, total protein, lipids and nitrogen composition. Briefly, water content was determined by drying *D. gigas* endoskeletons at 105 °C until a constant weight was reached. Ash content and total nitrogen were quantified following the AOAC protocol [48]. Total protein content in the solid samples was estimated as the sum of the amino acids analyzed using the ninhydrin reaction method with an amino acid analyzer [49]. The Bligh and Dyer method [47] was used to extract and determine the total lipids. Additionally, the content of heavy metals (Hg, Cd, and Pb) was analyzed using inductively coupled plasma mass spectrometry (ICP-MS). Chitin content was calculated by subtracting the weight of ash, protein, and lipids from the initial weight of dried pens. This approach is commonly used in pilot-scale and industrial studies due to its simplicity and reproducibility, particularly when working with relatively pure sources such as squid pens, which contain minimal mineral and pigment content. While more direct analytical methods such as spectroscopic assays may offer higher precision, the subtraction method has been validated in previous studies [50,51] and is considered sufficiently accurate for the scope of this work.

### 3.2. Chitin Extraction

Deproteinization treatment was performed by enzymatic hydrolysis to recover chitin from the industrial fishery discards of *D. gigas*. Ground squid pens were treated in duplicate with two commercial enzymes, Novozym 370731 and Alcalase 2.4 L, from Novozymes A/S (Novodirsk, Bagsvaerd, Denmark), at two different concentrations: 0.25% (CHN 25 and CHA 25) and 0.5% (volume of enzyme/weight of squid pens) (CHN 50 and CHA 50). Reaction with Alcalase was conducted at 55 °C and pH 9.0, while proteolysis with Novozym was performed at 60 °C and pH 8.5. All other reaction conditions were kept constant across the eight independent experiments: solid:liquid ratio of 1:10, agitation at 200 rpm, and reaction time of 6 h. The kinetics of hydrolysis were monitored using a controlled pH-Stat system with a 100 mL glass-reactor, following previously reported procedures [16]. At the end of the proteolysis, chitin (solid fraction) was separated from protein-rich effluents by centrifugation (3273× *g* for 5 min), followed by filtration, drying at 80 °C, and storage at room temperature. The collected protein effluents were frozen until further analysis. Proximate composition and chitin content were determined as specified in Section 3.1.

### 3.3. Chitin Deacetylation to Chitosan

Chitin obtained through deproteinization with Novozym (CHN 25 and CHN 50) and Alcalase (CHA 25 and CHA 50) was pooled into two batches, one for each enzyme. Each batch was then divided into three portions and subjected to partial deacetylation to produce chitosan via alkaline treatment at 90 °C under three different conditions: (i) 12.5 M NaOH for 12 h, yielding CSA1 (from Alcalase-treated chitin) and CSN1 (from Novozym-treated chitin); (ii) 15 M NaOH for 6 h, producing CSA2 (from chitin deproteinized with Alcalase) and CSN2 (from chitin deproteinized with Novozym); and 15 M NaOH for 24 h, generating CSA3 (from chitin deproteinized with Alcalase) and CSN3 (from chitin deproteinized with Novozym). Reactions were carried out in a round bottom flask under reflux and continuous magnetic stirring at a solid:liquid ratio of 1:10. Upon completion, the reaction mixtures were cooled in an ice bath, filtered through sintered glass (100–160 µm), and washed with water until neutral pH was reached. The produced chitosan was then dried in an oven at 80 °C overnight.

### 3.4. Chitosan Purification and Depolymerization

Based on the deproteinization and deacetylation results, treatment with Novozym at 0.5% (*w*/*v*) followed by reaction with 15 M NaOH for 24 h was selected for the upscaled production of *D. gigas* native chitosan (CS0) at a reactor volume of 5 L. The effluents generated from deproteinization (CE) and deacetylation (ChE) were frozen at −18 °C until further use as protein substrates for probiotics production. *D. gigas* native chitosan was purified by overnight dissolution in 1% (*w*/*w*) acetic acid at a concentration of 10 g/L, followed by filtration and precipitation using a methanol:30% ammonia solution at a 1:10 (*v*/*v*) ratio. After cooling at 4 °C for 1 h, the mixture was centrifuged in 1 L bottles at 13,261× *g* for 20 min and 4 °C on a Beckman Coulter Avanti J-25I centrifuge (Beckman Coulter, Inc, Indianapolis, IN, USA). The resulting chitosan (CS0) was then washed three times with water, followed by a final acetone wash, dried overnight at 60 °C, and freeze-dried.

Purified CS0 was subjected to depolymerization with sodium nitrite to obtain derivatives of different molecular weight [52] at three levels: high (CSH), medium (CSM), and low (CSL). After dissolving CS0 overnight in 0.05 M HCl at a concentration of 8 g/L, the depolymerization reaction was carried out at room temperature under continuous stirring by adding the right amount of a 1.6 g/L sodium nitrite solution, calculated according to the following equation:

$$n=\left(\frac{1}{M_{w}^{f}}+\frac{1}{M_{w}^{0}}\right)w$$

where n represents the moles of nitrite, $M_{w}^{f}$ the target molecular weight, $M_{w}^{0}$ the initial molecular weight, and w the weight of chitosan in the solution. The reaction kinetics was monitored by injecting aliquots of the reaction mixture onto the Gel Permeation Chromatography (GPC) system below described until a stable Mw was observed. This procedure was repeated until the target molecular weight was achieved. Chitosan was then precipitated with 10 M NaOH, centrifuged at 16,783× *g* for 20 min, and the pellet was washed with distilled water followed by successive centrifugations until neutrality. Finally, chitosan was oven-dried.

### 3.5. Polymer Characterization

The molecular weight of the native chitosan and its derivatives was determined by GPC using an Agilent 1260 liquid chromatography (LC) system (Agilent, Santa Clara, CA, USA) equipped with a quaternary pump (G1311B), autosampler (G1329B), column oven (G1316A), refractive index (G1362A), and dual angle static light scattering (G7800A) detectors. Chitosan separation was achieved using a set of four columns: Novema Max Precolumn (10 mm, 8 × 50 mm), Novema Max 30 Å (10 mm, 8 × 300 mm), Novema Max 1000 Å (10 mm, 8 × 300 mm) and Novema Max 1000 Å (10 mm, 8 × 300 mm) from Polymer Standards Service (PSS, Mainz, Germany) kept at 30 °C. Samples were eluted with a 0.3% (*v*/*v*) formic acid—0.1 M sodium chloride solution pumped at 1 mL/min after a 100 μL injection. Chitosan samples were dissolved in the GPC buffer at a concentration of 2 g/L. The detectors were calibrated using a polyethylene oxide standard (PSS, Mainz, Germany) of 106 kDa (Mp) and a polydispersity index of 1.05. The absolute Mw of chitosan was estimated using a refractive index increment, dn/dC, value of 0.18 [53]. Prior to injection, all chitosan samples and standards were filtered through 0.2 µm polyethersulfone (PES) syringe filters.

The deacetylation degree (DD) was determined by Proton Nuclear Magnetic Resonance (^1^H-NMR), performed at room temperature on a Bruker Avance Neo spectrometer (Billerica, MA, USA) at a resonance frequency of 400 MHz. Spectral processing was carried out using Mestrenova 10.0 software (Mestrelab Research, Santiago de Compostela, Spain). Chitosan samples were dissolved in 0.056 M deuterated trifluoroacetic acid (TFA-d in D2O) at a concentration of 8 g/L. The DD values were calculated based on the relative integrals of acetyl (N-acetyl and AcOH) and combined H2-H6 proton (glucosamine and N-acetylglucosamine, GluN and GluNAc, respectively) signals [54,55].

### 3.6. Chemical Analysis of Residual Effluents

In the protein and alkaline effluents generated in the optimal conditions for chitin isolation (CE) and chitosan production (ChE), the concentration of soluble protein [56], total sugars [57], the profile of amino acids, and the content of reducing sugars [58] were determined. All analytical determinations were made in duplicate. The composition of both effluents CE and ChE is summarized in Tables S1 and S2 (Supplementary Materials).

### 3.7. Antimicrobial Activity

Strains of *Escherichia coli* (CECT 102) and *Bacillus cereus* (CECT 495) were obtained from Colección Española de Cultivos Tipo (CECT, Universidad de Valencia, Valencia, Spain). Stock cultures were cryopreserved at −80 °C in 25% glycerol-supplemented meat-peptone broth (MPB) containing 0.5 g/L meat extract (Scharlau SL, Barcelona, Spain), 10 g/L (*w*/*v*) bacteriological peptone (BactoTM, BD Biosciences, Franklin Lakes, NJ, USA) and 5 g/L NaCl (Emsure R, Merck KGaA, Darmstadt, Germany).

Stock solutions of each chitosan were prepared at a concentration of 10 g/L by dissolving the chitosan in 0.1% (*v*/*v*) acetic acid, followed by sterilization via autoclaving at 121 °C for 15 min. Molecular weight analysis by GPC before and after autoclaving confirmed negligible changes in chitosan molecular weight (Figure S1). From these stock solutions, a 1:10 dilution was prepared in MPB, followed by serial 1:2 dilutions in 0.1% acetic acid MPB, yielding a final chitosan concentration range of 3.9–500 mg/L. The pH of each dilution was adjusted to 6.2 using 2 M NaOH at room temperature (25 °C) under continuous agitation at 180 rpm. It was observed that increasing the pH to 6.4 resulted in chitosan precipitation, regardless of the molecular weight.

The growth kinetics assay was performed following the guidelines of the Clinical and Laboratory Standards Institute (CLSI) for dilution antimicrobial susceptibility tests, with some modifications [59]. Briefly, 180 μL of each chitosan dilution was transferred to microplate wells. The same volume of 0.1% acetic acid MPB was employed as a control. Wells were subsequently inoculated with 20 μL of an overnight bacterial culture in 0.1% acetic acid MPB, adjusted to a final concentration of 1–2 × 10^8^ CFU/mL. For B. cereus, the presence of spores in the inoculum was assessed following the methodology of [60]. Results showed negligible spore concentration, below 1 × 10^5^% respecting to the *B. cereus* vegetative cells population.

The final acetic acid concentration in each well was 0.1%, and the final cell concentration was approximately 5 × 10^5^ CFU/mL. Bacterial growth was spectrophotometrically monitored at 600 nm at 30 min intervals over 24 h using a microplate reader (Multiskan Spectrum, Thermo Scientific, Waltham, MA, USA). Assays were conducted under agitation (180 rpm) and 30 °C for *B. cereus* and 37 °C for *E. coli*. Each chitosan concentration was tested in quadruplicate for both bacterial strains. The Minimum Inhibitory Concentration (MIC) was defined as the lowest chitosan concentration that prevents visible bacterial growth after 24 h [59].

Additionally, the values of optical density (*OD_m_*) at each concentration of chitosan (*OD_m_*_,*ch*_) were used to calculate the percentages of the inhibition of both bacteria in relation to the *OD_m_* without chitosan (*OD_m_*_,0_) according to the next equation:

$$I=100\times \left(1-\frac{{OD}_{m,ch}}{{OD}_{m,0}}\right)$$

Later on, the inhibition-concentration (*IC*) of chitosan relationships were evaluated and modelled by means of the Weibull equation [61] in order to obtain the value of the half maximal inhibitory concentration (*IC*_50_):

$$I=I_{m}\left\{1-\exp\left[-\ln2{\left(\frac{C}{{IC}_{50}}\right)}^{a}\right]\right\}$$

where *I* is the inhibition of the bacteria growth (%), *C* is the concentration of chitosan (mg/L), *I_m_* is the maximum inhibition (%), a is a form parameter (dimensionless) and *IC*_50_ is the half maximal inhibitory concentration (mg/L).

### 3.8. Residual Effluents Bioconversion by Probiotic Bacteria

Four bacteria were used to validate the CE and ChE bioconversion process: two lactic acid bacteria (LAB) from CECT (Spanish Type Culture Collection) with probiotic properties for humans (*Lactobacillus plantarum* CECT 220, Lb, and *Leuconostoc mesenteroides* CECT 4046, Ln), and two marine bacteria (MB) with recognized probiotic effect in aquaculture (*Phaeobacter* sp., Pha, and *Pseudomonas fluorescens*, Pf). Stock cultures were stored at −80 °C in Man, Rogosa and Sharpe medium (MRS, from Condalab, Torrejón de Ardoz, Madrid, Spain) for LAB, and Marine medium (MM, from Difco, MD, USA) for MB, both supplemented with 25% glycerol (*w*/*w*).

For LAB, four low-cost media formulations using CE and ChE as basic protein sources were evaluated (Table S3, Supplementary Materials): (a) MRS-like broths where commercial peptones (meat extract and bactopeptone) were replaced by ChE (medium D) and CE (medium C); (b) minima media similar to the previous formulations but without yeast extract (medium B); (c) minima media containing only glucose and CE (medium A). Mineral salts and Tween 80 were purchased from Sigma-Aldrich (Burlington, MA, USA), glucose was supplied by Vorquímica S.L. (Vigo, Spain), and yeast extract was obtained from Panreac Applichem (Barcelona, Spain). For all formulations, the initial glucose concentration (reducing sugars) was adjusted to 20–22 g/L, and the pH was set to 6.0 using 5N NaOH. Media were sterilized separately at 121 °C for 15 min. Fermentations were carried out in triplicate at 30 °C and 200 rpm in an orbital shaker (New Brunswick Innova^®^ 43/43R, Edison, NJ, USA) using 300 mL Erlenmeyer flasks with 200 mL working volume. Inocula (0.5%, *v*/*v*) were prepared from 12–16 h LAB cultures in MRS medium.

For MB, alternative media containing CE and ChE were formulated (Table S4, Supplementary Materials): (a) medium with CE and seawater (medium A); (b) medium with ChE and seawater (medium B); (c) medium with CE, yeast extract and seawater (medium C); (d) medium with ChE, yeast extract and seawater (medium D). The initial pH was adjusted to 7.5 with 5N NaOH, and media were sterilized separately at 121 °C for 15 min. Fermentations were performed in triplicate at 22 °C and 200 rpm in an orbital shaker (New Brunswick Innova^®^ 43/43R, Edison, NJ, USA) using 300 mL Erlenmeyer flasks with 150 mL working volume. Inocula (0.5%, *v*/*v*) were prepared from 12–16 h bacterial cultures in MM.

At pre-defined time points, aliquots were collected from each flask and centrifuged at 3273× *g* for 15 min (Allegra X-12R centrifuge, Beckman-Coulter, Brea, CA, USA). Supernatants were analyzed for biomass production (both bacterial types), organic acid (lactic and acetic) production in LAB, glucose consumption (in LAB) and soluble protein (in LAB and MM). The pellet was washed, resuspended in distilled water, and appropriately diluted for optical density (OD) measurements at 700 nm (UV/Vis Lambda 365 spectrophotometer, Perkin Elmer, Waltham, MA, USA), with dry weight estimated using a calibration curve (OD vs. dry weight). In LAB-free supernatants, the total soluble protein was quantified using the Lowry method [56], glucose as reducing sugars was measured by 3,5-dinitrosalicylic reaction [58], and organic acids were analyzed using a Beckman system gold HPLC (Brea, CA, USA) equipped with a refractive index detector (Thermo, Waltham, MA, USA) and ION-300 column (Transgenomic, San José, CA, USA) [62]. In MB supernatants, only total soluble protein was analysed [56].

The experimental dynamic data on bacterial growth, nutrient consumption, and lactic acid production were simulated using an unstructured mathematical model, as summarized in Equations [1–6] and detailed in Tables S5 and S6 (Supplementary Materials) [25].

### 3.9. Numerical Fitting and Statistical Analyses

Fitting procedures of experimental data and parametric estimations (the biological meaning of parameters are summarized in Table S6, Supplementary Materials) were calculated by minimizing the sum of quadratic differences between observed and model predicted values, using the non-linear least-squares (GRG non-linear) method available in the macro-‘Solver’ of the Microsoft Excel 2016 spreadsheet. Confidence intervals for parametric estimates were determined using Student’s *t* test, while the consistency of mathematical models was assessed through Fisher’s F test, both evaluated using the “SolverAid” macro. A one-way ANOVA test followed by means of Tukey test was applied to identify significant differences between fermentation parameters across the tested growth media. This procedure was also used to evaluate differences in chitosan antibacterial effects. Statistical significance was set at *p* < 0.05.

## 4. Conclusions

This study presents a sustainable and integrated biorefinery approach for the valorization of *Dosidicus gigas* squid pen waste. Enzymatic deproteinization with both Alcalase and Novozym proves effective, though Novozym demonstrates slightly superior efficiency, yielding a chitin-rich material (73%). The extracted chitin undergoes then subsequent alkaline treatment and controlled depolymerization to render highly deacetylated chitosan (>90% degree of deacetylation) with varying molecular weights (50–251 kDa). Both native and depolymerized chitosan exhibit antimicrobial activity against *E. coli* and *B. cereus*, with *B. cereus* displaying greater resistance. Critically, this work repurposes the protein-rich effluents generated during both deproteinization and deacetylation processes as nitrogen sources for the cultivation of human (*L. plantarum* and *L. mesenteroides*) and marine (*Pseudomonas fluorescens* and *Phaeobacter* sp.) probiotics. Substituting peptones with these effluents in commercial media results in comparable growth and lactic acid production in human probiotic cultures, with the deproteinization effluent proving more effective than the deacetylation effluent. Although both residual streams support marine probiotic growth, it is less pronounced in the alternative media. This integrated biorefinery approach enables the conversion of 1 kg of squid pens into 350 g of chitosan. While lactic acid production reached 854–856 g using human lactic acid bacteria cultures, it is important to note that this yield is primarily dependent on the addition of 1.2 kg of glucose to the fermentation media, which is not derived from squid pens. Therefore, lactic acid output should be interpreted in the context of glucose supplementation rather than as a direct conversion from squid pen biomass. This process maximizes resource utilization, minimizes waste generation, and produces valuable products, contributing to a more sustainable and circular seafood industry. Scaling up these processes is essential for commercial viability and widespread implementation.

## Figures and Tables

**Table 1:** Deacetylation degree (%) of chitosan samples after reaction at 90 °C.

		DD%		DD%
12.5 M NaOH 12 h	CSN1	85.9	CSA1	87.0
15 M NaOH 6 h	CSN2	87.2	CSA2	86.2
15 M NaOH 24 h	CSN3	91.5	CSA3	90.7

**Table 2:** Molecular weight of chitosan obtained after deproteinization with 0.5% Novozym and deacetylation with 15 M NaOH at 90 °C for 24 h and depolymerized derivatives. Mn: Number average molecular weight; Mw: Weight average molecular weight (mean ± standard deviation, *n* = 2); PDI = Mw/Mn (polydispersity index).

	Mn (kDa)	Mw (kDa)	PDI	Retention Time (min)
CS0	251 ± 18	389 ± 4	1.549	18.26
CSH	137 ± 9	196 ± 5	1.432	18.81
CSM	94 ± 11	139 ± 7	1.484	19.19
CSL	50 ± 6	82 ± 6	1.649	20.12

## Data Availability

Data will be made available upon request.

## Supplementary Materials

The following supporting information can be downloaded at: https://www.mdpi.com/article/10.3390/md23100382/s1, Table S1. Amino acids (AA) content of squid pen (SP), chitin isolated (Ci), chitosan (Ch), chitin effluent (CE) and chitosan effluent (ChE) (% or g/100 g total amino acids). OHPro: hydroxyproline. TEAA/TAA: ratio of total essential amino acids for human/total amino acids as percentage. TP: total protein content as percentage of solid sample. Errors are the confidence intervals for *n* = 2 (replicates of two analysis) and α = 0.05; Table S2. Chemical composition of the effluents generated in the production of chitin and chitosan from *Dosidicus gigas* squid pens. CE: residual effluent from the desproteinization (enzymatic hydrolysis) of pens. ChE: residual effluent from alkaline deacetylation of chitin. Pr: soluble proteins. TS: total sugars. RS: reducing sugars. Errors are the confidence intervals for *n* = 3 y α = 0.05.; Table S3. Composition of the culture media (in g/L) used for the fermentation of LAB probiotics. Medium A: culture medium formulated with glucose and chitin effluent (CE). Medium B: culture medium formulated with glucose, mineral salts and chitin effluent (CE). Medium C: culture medium similar to MRS in which commercial peptones were replaced by CE. Medium D: culture medium similar to MRS in which commercial peptones were replaced by chitosan effluent (ChE). Initial pH: 6.2. Sterilization: 121 °C/15 min.; Table S4. Composition of the culture media (in g/L) used for the fermentation of marine probiotics. Medium A: culture medium formulated with seawater and chitin effluent (CE). Medium B: culture medium formulated with seawater and chitosan effluent (ChE). Medium C: culture medium formulated with seawater, yeast extract and chitin effluent (CE). Medium D: culture medium formulated with seawater, yeast extract and chitosan effluent (ChE). * Volume of filtrated and sterilized seawater needed for residual media preparation. ** Volume of distilled water needed for commercial medium preparation. Initial pH: 7.5. Sterilization: 121 °C/15 min; Table S5. Mathematical equations (unstructured model) used for probiotics fermentation modelling; Table S6. Parameter definitions (symbolic notations) and corresponding units of the unstructured model shown in Table S5; Table S7. Numerical values and confidence intervals for parameters obtained from experimental data of *E. coli* growth on culture media formulated with various concentrations of chitosan from different molecular weights. Experimental data were fitted to the logistic Equation [1]. R_2_ are the determination coefficients among experimental and predicted data. Different letters in each column (as superscript) mean significant differences between media (*p* < 0.05). ODm: maximum optical density. VOD: maximum optical density rate. λOD: lag phase of optical density; Table S8. Numerical values and confidence intervals for parameters obtained from experimental data of *B. cereus* growth on culture media formulated with various concentrations of chitosan from different molecular weights. Experimental data were fitted to the logistic Equation [1]. R_2_ are the determination coefficients among experimental and predicted data. Different letters in each column (as superscript) mean significant differences between media (*p* < 0.05). ODm: maximum optical density. VOD: maximum optical density rate. λOD: lag phase of optical density; Table S9. Values of IC50 (as %) for each type of chitosan studied; Table S10. Numerical values and confidence intervals for parameters obtained from mathematical modelling of experimental data of L. plantarum (X, La, G and Pr), grown on media summarized in Table S3, by Equations [1–6]. R_2_ are the determination coefficients among experimental and predicted data. *p*-value was estimated by F-Fisher test. Different letters in each column indicate a significant difference between media (*p* < 0.05). NS: not significant; Table S11. Numerical values and confidence intervals for parameters obtained from mathematical modelling of experimental data of *L. mesenteroides* (X, La, G and Pr), grown on media summarized in Table S3, by Equations [1–6]. R_2_ are the determination coefficients among experimental and predicted data. *p*-value was estimated by F-Fisher test. Different letters in each column indicate a significant difference between media (*p* < 0.05). NS: not significant; Table S12. Numerical values and confidence intervals for parameters obtained from mathematical modelling of the experimental data from *P. fluorescens* and *Phaeobacter* sp. (X and Pr), grown on media summarized in Table S4, by Equations [1–6]. R_2_ are the determination coefficients among experimental and predicted data. *p*-value was estimated by F-Fisher test. Different letters in each column indicate a significant difference between media (*p* < 0.05). NS: not significant; Table S13. Summary Table of Mass Yields per kg of Squid Pens Treated. *L. plantarum*: Lb; *L. mesenteroides* (Ln); Figure S1. Gel permeation chromatography eluogram (right angle light scattering signal) of pre-autoclave and post-autoclave chitosan (251 kDa). Very close match of peaks evidence a negligible autoclaving (121 °C, 15 min) effect on chitosan molecular weight. Please refer to Section 2.5. Polymer characterization for details about the analytical details; Figure S2. Dose-response relationships between the concentration of chitosan and the growth inhitibition (in terms of ODm) of *E. coli* (left) and *B. cereus* (right) for each chitosan molecular weights studied. These DR profiles are fitted to the Weibull equation (lines); Figure S3. Biomass dynamics, lactic acid productions and nutrients uptakes for Lb growing in the media discribed in Table S3. Experimental data of biomass (X), lactic acid (La), proteins (Pr) and glucose (G) were fitted by the equations displayed in Table S5 (continuous lines). Medium A (●), medium B (●), Medium C (●), Medium D (●) and MRS (●). Error bars represent the confidence intervals for *n* = 3 and α = 0.05; Figure S4. Culture kinetics of Pf (top) and Pha (bottom) in the media discribed in Table S4. Experimental data of biomass (X) and proteins (Pr) were fitted by the equations displayed in Table S5 (continuous lines). Medium A (●), Medium C (●), Medium D (●) and MM (●). Error bars represent the confidence intervals for *n* = 3 and α = 0.05.
