# Supplementary material for: Sustainable and Integral Valorization of Dosidicus gigas Pen Waste: Combined Production of Chitosan with Antibacterial Properties and Human and Marine Probiotics
Source: Mar Drugs. 2025 Sep 27;23(10):382. doi: 10.3390/md23100382 (PMC12565315; doi:10.3390/md23100382)
Supplement: Supplementary file 1 [file marinedrugs-23-00382-s001.zip › marinedrugs-3884835- Supplementary material.pdf]

SUPPLEMENTARY MATERIAL

Sustainable and integral valorization of *Dosidicus gigas* pen wastes: Combined production of chitosans with antibacterial properties and human and marine probiotics

Marta Lima [1], Adrián Pedreira [2,3], Noelia Sanz [2], José Antonio Vázquez [2], Míriam R. García [3], Filipe Mergulhão [1] and Jesus Valcarcel [2,4,*]

[1] LEPABE, ALiCE, Faculty of Engineering, University of Porto, Rua Dr. Roberto Frias, 4200-465 Porto, Portugal.; up201604683@edu.fe.up.pt (M.L.); filipem@fe.up.pt (F.M.)

[2] Recycling and Valorization of Waste Materials (REVAL), Marine Research Institute (IIM-CSIC), Eduardo Cabello 6, 36208 Vigo, Spain; apedreira@iim.csic.es (A.P.); nsanz@iim.csic.es (N.S.); jvazquez@iim.csic.es (J.A.V.)

[3] Bioprocess Engineering (Bio2eng), Marine Research Institute (IIM-CSIC), Eduardo Cabello 6, 36208 Vigo, Spain; miriamr@iim.csic.es

[4] Scientific Technical Support Unit (UACT), Marine Research Institute (IIM-CSIC), Eduardo Cabello 6, 36208 Vigo, Spain

Table S1. Amino acids (AA) content of squid pen (SP), chitin isolated (Ci), chitosan (Ch), chitin effluent (CE) and chitosan effluent (ChE) (% or g/100 g total amino acids). OHPro: hydroxyproline. TEAA/TAA: ratio of total essential amino acids for human/total amino acids as percentage. TP: total protein content as percentage of solid sample. Errors are the confidence intervals for n=2 (replicates of two analysis) and α=0.05.

AA	SP	Ci	Ch	CE	ChE
**Asp**	7.28±0.03	3.87±0.01	0.05±0.01	6.92±0.07	3.94±0.02
**Thr**	1.71±0.12	2.32±0.01	0.00	3.70±0.04	0.91±0.12
**Ser**	2.85±0.00	2.04±0.01	0.04±0.00	3.29±0.06	0.79±0.04
**Glu**	3.23±0.06	2.11±0.07	0.06±0.00	4.62±0.03	2.84±0.04
**Gly**	11.54±0.04	5.92±0.33	0.00	8.30±0.03	4.37±0.18
**Ala**	11.01±0.20	6.50±0.53	0.00	9.82±0.08	8.75±0.13
**Cys**	0.77±0.03	1.24±0.13	0.20±0.02	2.54±0.04	0.69±0.13
**Val**	3.11±0.08	3.70±0.15	0.05±0.01	5.24±0.05	9.68±0.06
**Met**	1.11±0.06	1.55±0.01	0.06±0.01	1.44±0.05	3.23±0.41
**Ile**	2.13±0.03	2.42±0.01	0.05±0.00	2.82±0.14	5.11±0.06
**Leu**	3.75±0.04	4.25±0.11	0.15±0.00	6.42±0.01	10.75±0.14
**Tyr**	25.63±0.39	43.53±1.23	96.56±0.58	14.76±0.03	13.93±0.23
**Phe**	3.90±0.05	2.14±0.04	0.44±0.09	3.10±0.09	6.34±0.08
**His**	9.32±0.05	8.42±0.42	0.90±0.19	10.74±0.14	7.67±0.16
**Lys**	4.47±0.04	2.68±0.03	0.70±0.13	3.92±0.07	5.76±0.19
**Arg**	1.19±0.06	1.43±0.04	0.08±0.01	2.13±0.06	2.36±0.28
**OHPro**	0.18±0.04	0.05±0.02	0.00	0.12±0.01	0.00
**Pro**	6.83±0.04	5.64±0.12	0.65±0.14	10.11±0.40	12.89±0.26
**TEAA/TAA (%)**	30.7±0.6	28.9±0.4	2.4±0.4	39.5±0.3	51.8±0.8
**TP (%)**	52.7±4.9	24.8±3.6	3.1±0.9	-	-

Table S2. Chemical composition of the effluents generated in the production of chitin and chitosan from *Dosidicus gigas* squid pens. CE: residual effluent from the desproteinization (enzymatic hydrolysis) of pens. ChE: residual effluent from alkaline deacetylation of chitin. Pr: soluble proteins. TS: total sugars. RS: reducing sugars. Errors are the confidence intervals for n= 3 y α= 0.05.

Effluent	Pr (g/L)	TS (g/L)	RS (g/L)
**CP**	59.30 ± 22.40	0.13 ± 0.08	0.10 ± 0.04
**ChP**	3.40 ± 0.90	0.09 ± 0.04	0.09 ± 0.07

Table S3. Composition of the culture media (in g/L) used for the fermentation of LAB probiotics. Medium A: culture medium formulated with glucose and chitin effluent (CE). Medium B: culture

medium formulated with glucose, mineral salts and chitin effluent (CE). Medium C: culture medium similar to MRS in which commercial peptones were replaced by CE. Medium D: culture medium similar to MRS in which commercial peptones were replaced by chitosan effluent (ChE). Initial pH: 6.2. Sterilization: 121ºC/15 min.

INGREDIENTS	Medium A	Medium B	Medium C	Medium D	MRS
**Glucose**	20	20	20	20	24
**Yeast extract**	-	-	4	4	4
**Sodium acetate**	-	5	5	5	5
**Ammonium citrate**	-	2	2	2	2
**K_2HPO_4**	-	2	2	2	2
**$MgSO_4$**	-	0.2	0.2	0.2	0.2
**$MnSO_4$**	-	0.05	0.05	0.05	0.05
**Tween 80**	-	1	1	1	1
**Meat extract**	-	-	-	-	8
**Bactopeptone**	-	-	-	-	10
**Effluents (Lowry-Protein)**	10	10	10	3.4	-

Table S4. Composition of the culture media (in g/L) used for the fermentation of marine probiotics. Medium A: culture medium formulated with seawater and chitin effluent (CE). Medium B: culture medium formulated with seawater and chitosan effluent (ChE). Medium C: culture medium formulated with seawater, yeast extract and chitin effluent (CE). Medium D: culture medium formulated with seawater, yeast extract and chitosan effluent (ChE). *Volume of filtrated and sterilized seawater needed for residual media preparation. **Volume of distilled water needed for commercial medium preparation. Initial pH: 7.5. Sterilization: 121ºC/15 min.

INGREDIENTS	Medium A	Medium B	Medium C	Medium D	MM
**Ferric citrate**	-	-	-	-	0.10
**Sodium chloride**	-	-	-	-	19.45
**Magnesium chloride**	-	-	-	-	5.90
**Sodium sulphate**	-	-	-	-	3.24
**Calcium chloride**	-	-	-	-	1.80
**Potassium chloride**	-	-	-	-	0.55
**Sodium bicarbonate**	-	-	-	-	0.16
**Potassium bromide**	-	-	-	-	0.08
**Strontium chloride**	-	-	-	-	34 mg
**Boric acid**	-	-	-	-	22 mg
**Sodium silicate**	-	-	-	-	4 mg
**Sodium fluoride**	-	-	-	-	2.4 mg
**Ammonium nitrate**	-	-	-	-	1.6 mg
**Disodium phosphate**	-	-	-	-	8 mg
**Yeast extract**	-	-	1	1	1
**Neopeptone**	-	-	-	-	5
**Effluents (Lowry-Protein)**	2.6	2.6	2.6	2.6	-
**Seawater (L)***	1	1	1	1	-
**Distilled water (L)****	-	-	-	-	1

Table S5. Mathematical equations (unstructured model) used for probiotics fermentation modelling.

$X=\dfrac{X_m}{1+\exp\left[2+\dfrac{4v_X}{X_m}(\lambda_X-t)\right]}$ with $X_0=\dfrac{X_m}{1+\exp\left(2+\dfrac{4v_X\lambda_X}{X_m}\right)}$	[1]
$L_a=\dfrac{L_m}{1+\exp\left[2+\dfrac{4v_L}{L_m}(\lambda_L-t)\right]}$ with $L_{a0}=\dfrac{L_m}{1+\exp\left(2+\dfrac{4v_L\lambda_L}{L_m}\right)}$	[2]
$G=G_0-\dfrac{1}{Y_{x/g}}\left[\dfrac{X_m}{1+\left(\dfrac{X_m}{X_0}-1\right)\exp\left(-\dfrac{4v_X}{X_m}t\right)}-X_0\right]-\left(\dfrac{m_g X_m^2}{4v_X}\right)\ln\left[\dfrac{X_0\left(e^{\frac{4v_X}{X_m}t}-1\right)+X_m}{X_m}\right]$	[3]
$Pr=Pr_0-\dfrac{1}{Y_{x/p}}\left[\dfrac{X_m}{1+\left(\dfrac{X_m}{X_0}-1\right)\exp\left(-\dfrac{4v_X}{X_m}t\right)}-X_0\right]-\left(\dfrac{m_p X_m^2}{4v_X}\right)\ln\left[\dfrac{X_0\left(e^{\frac{4v_X}{X_m}t}-1\right)+X_m}{X_m}\right]$	[4]
$L_a=L_{a0}+Y_{L/x}\left[\dfrac{X_m}{1+\left(\dfrac{X_m}{X_0}-1\right)\exp\left(-\dfrac{4v_X}{X_m}t\right)}\right]-Y_{L/x}X_0$	[5]
$L_a=\dfrac{-X_0Y_{L/g}}{Y_{x/g}}+\dfrac{Y_{L/g}}{Y_{x/g}}\left[\dfrac{X_m}{1+\left(\dfrac{X_m}{X_0}-1\right)\exp\left(-\dfrac{4v_X}{X_m}t\right)}\right]+\left(\dfrac{m_g X_m^2 Y_{L/g}}{4v_X}\right)\ln\left[\dfrac{X_0\left(e^{\frac{4v_X}{X_m}t}-1\right)+X_m}{X_m}\right]$	[6]

Table S6. Parameter definitions (symbolic notations) and corresponding units of the unstructured model shown in Table S5.

X :	Biomass. Unit: g/L
t :	Time. Unit: h
X_m :	Maximum biomass. Unit: g/L
X_0 :	Initial biomass. Unit: g/L
v_X :	Maximum growth (biomass production) rate. Unit: g $L^{-1}h^{-1}$
λ_X :	Growth lag phase. Unit: h
L_a :	Lactic acid. Unit: g/L
L_{a0} :	Initial lactic acid. Unit: g/L
L_m :	Maximum lactic acid production. Unit: g/L
v_L :	Maximum rate of lactic acid production. Unit: g $L^{-1}h^{-1}$
λ_L :	Lactic acid lag phase. Unit: h
G :	Glucose. Unit: g/L
G_0 :	Initial glucose. Unit: g/L
$Y_{x/g}$:	Yield factor for biomass formation on glucose. Unit: g biomass / g glucose
m_g :	Maintenance coefficient for glucose. Unit: g (glucose) g^{-1} (biomass) h^{-1}
$Y_{L/g}$:	Yield factor for lactic acid production per glucose consumed. Unit: g lactic acid / g glucose
$Y_{L/x}$:	Yield factor for lactic acid formation per biomass produced. Unit: g lactic acid / g biomass
Pr :	Soluble Protein. Unit: g/L
Pr_0 :	Initial soluble protein. Unit: g/L
$Y_{x/p}$:	Yield factor for biomass formation on protein. Unit: g biomass / g protein
m_p :	Maintenance coefficient for protein. Unit: g (protein) g^{-1} (biomass) h^{-1}

Table S7. Numerical values and confidence intervals for parameters obtained from experimental data of *E. coli* growth on culture media formulated with various concentrations of chitosan from different molecular weights. Experimental data were fitted to the logistic equation [1]. R^2 are the determination coefficients among experimental and predicted data. Different letters in each column (as superscript) mean significant differences between media ($p<0.05$). OD_m: maximum optical density. V_{OD}: maximum optical density rate. λ_{OD}: lag phase of optical density.

	**40 kDa**					**90 kDa**				
	**0 mg/L**	**3.9 mg/L**	**7.8 mg/L**	**15.6 mg/L**	**31.2 mg/L**	**0 mg/L**	**3.9 mg/L**	**7.8 mg/L**	**15.6 mg/L**	**31.2 mg/L**
OD_m	0.539±0.004^{a}	0.288±0.003^{b}	0.268±0.003^{c}	0.216±0.006^{d}	-	0.539±0.004^{a}	0.293±0.004^{b}	0.264±0.004^{c}	0.247±0.004^{a}	-
v_{OD}	0.053±0.001^{a}	0.028±0.001^{b}	0.034±0.001^{c}	0.031±0.003^{b,c}	-	0.053±0.001^{a}	0.027±0.001^{b}	0.027±0.001^{b}	0.025±0.001^{b}	-
λ_{OD}	7.30±0.12^{a}	7.17±0.17^{a}	8.67±0.18^{b}	8.74±0.45^{b}	-	7.30±0.12^{a}	7.30±0.17^{a}	8.32±0.20^{b}	8.35±0.23^{b}	-
R^2_{OD}	0.999	0.999	0.998	0.990	-	0.999	0.999	0.998	0.998	-
	**130 kDa**					**251 kDa**				
OD_m	0.539±0.004^{a}	0.297±0.003^{b}	0.272±0.004^{c}	0.305±0.007^{b}	-	0.539±0.004^{a}	0.320±0.010^{b}	0.278±0.005^{c}	0.302±0.009^{b}	-
v_{OD}	0.053±0.001^{a}	0.030±0.001^{b}	0.031±0.001^{b}	0.044±0.003^{c}	-	0.053±0.001^{a}	0.028±0.002^{b}	0.028±0.001^{b}	0.035±0.003^{c}	-
λ_{OD}	7.30±0.12^{a}	7.39±0.15^{a}	8.40±0.21^{b}	11.23±0.30^{c}	-	7.30±0.12^{a}	7.16±0.42^{a}	8.28±0.24^{b}	10.68±0.34^{c}	-
R^2_{OD}	0.999	0.999	0.998	0.995	-	0.999	0.993	0.997	0.994	-

Table S8. Numerical values and confidence intervals for parameters obtained from experimental data of *B. cereus* growth on culture media formulated with various concentrations of chitosan from different molecular weights. Experimental data were fitted to the logistic equation [1]. R^2 are the determination coefficients among experimental and predicted data. Different letters in each column (as superscript) mean significant differences between media ($p<0.05$). OD_m: maximum optical density. V_{OD}: maximum optical density rate. λ_{OD}: lag phase of optical density.

	40 kDa					90 kDa				
	**0 mg/L**	**3.9 mg/L**	**7.8 mg/L**	**15.6 mg/L**	**31.2 mg/L**	**0 mg/L**	**3.9 mg/L**	**7.8 mg/L**	**15.6 mg/L**	**31.2 mg/L**
OD_m	$0.443±0.010^a$	$0.299±0.006^b$	$0.274±0.005^c$	$0.263±0.004^d$	-	$0.443±0.010^a$	$0.299±0.006^b$	$0.305±0.005^b$	$0.260±0.005^c$	-
v_{OD}	$0.044±0.004^a$	$0.040±0.004^a$	$0.031±0.002^b$	$0.032±0.002^b$	-	$0.044±0.004^a$	$0.040±0.004^a$	$0.040±0.003^a$	$0.027±0.002^b$	-
λ_{OD}	$4.25±0.45^a$	$4.55±0.40^a$	$4.63±0.38^a$	$4.51±0.30^a$	-	$4.25±0.45^a$	$4.81±0.44^{a,b}$	$5.00±0.31^b$	$5.47±0.34^b$	-
R^2_{OD}	0.991	0.991	0.993	0.995	-	0.991	0.989	0.994	0.995	-
	**130 kDa**					**251 kDa**				
OD_m	$0.443±0.010^a$	$0.291±0.004^b$	$0.264±0.005^c$	$0.247±0.004^d$	-	$0.443±0.010^a$	$0.319±0.005^b$	$0.253±0.004^c$	$0.250±0.005^c$	-
v_{OD}	$0.044±0.004^a$	$0.037±0.002^b$	$0.026±0.002^c$	$0.037±0.003^b$	-	$0.044±0.004^a$	$0.040±0.003^a$	$0.040±0.003^a$	$0.029±0.002^b$	-
λ_{OD}	$4.25±0.45^a$	$4.33±0.27^a$	$4.93±0.33^a$	$4.69±0.31^a$	-	$4.25±0.45^a$	$3.50±0.33^b$	$4.83±0.31^a$	$4.48±0.39^a$	-
R^2_{OD}	0.991	0.996	0.995	0.994	-	0.991	0.994	0.994	0.992	-

Table S9. Values of IC_{50} (as %) for each type of chitosan studied.

	50 kDa	94 kDa	137 kDa	250 kDa
E. coli	6.52	6.89	7.97	8.97
B. cereus	11.72	12.66	10.75	11.26

Table S10. Numerical values and confidence intervals for parameters obtained from mathematical modelling of experimental data of *L. plantarum* (*X*, *La*, *G* and *Pr*), grown on media summarized in Table S3, by equations [1-6]. R^2 are the determination coefficients among experimental and predicted data. p-value was estimated by F-Fisher test. Different letters in each column indicate a significant difference between media (p<0.05). NS: not significant.

Parameters	Medium A	Medium B	Medium C	Medium D	MRS
X_m (g/L)	0.14 ± 0.02^a	0.65 ± 0.07^b	2.28 ± 0.20^c	1.92 ± 0.12^d	2.40 ± 0.13^c
v_x (g $L^{-1}h^{-1}$)	0.006 ± 0.002^a	0.023 ± 0.004^b	0.148 ± 0.051^c	0.130 ± 0.032^c	0.176 ± 0.040^c
λ_x (h)	1.11 (NS)	10.6 ± 2.5^a	6.4 ± 2.9^a	6.5 ± 2.0^a	8.4 ± 1.7^a
R^2_X	0.982	0.988	0.986	0.992	0.995
p-value	< 0.0001	< 0.0001	< 0.0001	< 0.0001	< 0.0001
$Y_{X/g}$ (g X/g G)	0.057 (NS)	0.086 ± 0.041^a	0.163 ± 0.036^b	0.164 ± 0.034^b	0.146 ± 0.018^b
m_g (g X g^{-1} G h^{-1})	0.411 (NS)	0.156 (NS)	0.063 ± 0.015$^{a.b}$	0.085 ± 0.026^a	0.034 ± 0.024^b
$R^2_{X/g}$	0.656	0.967	0.992	0.996	0.992
p-value	< 0.05	< 0.001	< 0.001	< 0.001	< 0.001
$Y_{X/P}$ (g X/g Pr)	0.144 (NS)	0.441 ± 0.041^a	2.36 ± 0.52^b	2.42 ± 0.42^b	1.86 ± 0.37^b
m_P (g X g^{-1} Pr h^{-1})	-0.073 (NS)	-0.030 (NS)	0.004 ± 0.003^a	-0.001 (NS)	0.005 ± 0.003^a
$R^2_{X/P}$	0.522	0.817	0.912	0.971	0.974
p-value	< 0.05	< 0.001	< 0.001	< 0.001	< 0.001
L_m (g/L)	1.76 ± 1.74^a	7.76 ± 1.33^b	14.56 ± 1.57^c	13.72 ± 1.60^c	14.82 ± 0.85^c
v_L (g $L_a^{-1}h^{-1}$)	0.291 (NS)	0.239 ± 0.053^a	0.697 ± 0.222^b	0.651 ± 0.222^b	0.891 ± 0.185^b
λ_L (h)	20.1 ± 3.1^a	9.9 ± 3.5^b	6.0 ± 3.6^b	5.9 ± 3.8^b	7.1 ± 1.9^b
R^2_{La}	0.999	0.977	0.983	0.983	0.994
p-value	< 0.0001	< 0.0001	< 0.0001	< 0.0001	< 0.0001
$Y_{L/X}$ (g L_a/g X)	0.403 ± 0.131^a	0.821 ± 0.220^b	0.830 ± 0.259^b	0.874 ± 0.260^b	0.826 ± 0.219^b
$R^2_{La/X}$	0.820	0.981	0.994	0.995	0.989
p-value	< 0.05	< 0.0001	< 0.0001	< 0.0001	< 0.0001
$Y_{L/g}$ (g L_a/g G)	10.2 ± 1.9$^{a.b}$	11.1 ± 0.8^a	5.73 ± 1.64^b	6.31 ± 1.90^b	5.96 ± 1.28^b
$R^2_{La/g}$	0.662	0.982	0.969	0.962	0.989
p-value	< 0.05	< 0.001	< 0.001	< 0.001	< 0.0001

Table S11. Numerical values and confidence intervals for parameters obtained from mathematical modelling of experimental data of *L. mesenteroides* (*X*, *La*, *G* and *Pr*), grown on media summarized in Table S3, by equations [1-6]. R^2 are the determination coefficients among experimental and predicted data. p-value was estimated by F-Fisher test. Different letters in each column indicate a significant difference between media (p<0.05). NS: not significant.

Parameters	Medium A	Medium B	Medium C	Medium D	MRS
X_m (g/L)	0.18 ± 0.02^{a}	1.00 ± 0.11^{b}	3.65 ± 0.28^{c}	2.96 ± 0.24^{d}	3.96 ± 0.13^{c}
v_X (g $L^{-1}h^{-1}$)	0.006 ± 0.002^{a}	0.031 ± 0.005^{b}	0.245 ± 0.076$^{c.d}$	0.205 ± 0.071^{c}	0.353 ± 0.040^{d}
λ_X (h)	0.27 (NS)	8.6 ± 2.3^{a}	6.5 ± 2.6^{a}	6.4 ± 2.8^{a}	7.3 ± 1.7^{a}
R^2_X	0.979	0.990	0.987	0.985	0.994
p-value	< 0.0001	< 0.0001	< 0.0001	< 0.0001	< 0.0001
$Y_{X/g}$ (g X/g G)	0.079 ± 0.040^{a}	0.095 ± 0.016^{a}	0.218 ± 0.045^{b}	0.221 ± 0.050^{b}	0.222 ± 0.042^{b}
m_g (g X g^{-1} G h^{-1})	0.032 ± 0.017$^{a.b}$	-0.009 (NS)	0.025 ± 0.009^{a}	0.053 ± 0.012^{b}	0.001 ± 0.001^{c}
$R^2_{X/g}$	0.820	0.990	0.994	0.995	0.998
p-value	< 0.05	< 0.001	< 0.001	< 0.001	< 0.001
$Y_{X/P}$ (g X/g Pr)	0.485 (NS)	0.353 ± 0.201^{a}	1.77 ± 0.52^{b}	3.61 ± 0.78^{c}	2.62 ± 0.31^{c}
m_P (g X g^{-1} Pr h^{-1})	-0.007 (NS)	-0.073 (NS)	-0.003 (NS)	0.004 ± 0.002^{a}	0.002 ± 0.001^{a}
$R^2_{X/P}$	0.925	0.814	0.890	0.970	0.891
p-value	< 0.05	< 0.001	< 0.001	< 0.001	< 0.001
L_m (g/L)	1.87 ± 0.28^{a}	7.71 ± 1.15^{b}	14.39 ± 1.45^{c}	13.64 ± 1.60^{c}	13.04 ± 0.45^{c}
v_L (g $L_a^{-1}h^{-1}$)	0.081 ± 0.021^{a}	0.255 ± 0.057^{b}	0.729 ± 0.236^{c}	0.679 ± 0.228^{c}	1.073 ± 0.184^{c}
λ_L (h)	17.2 ± 3.0^{a}	9.6 ± 3.3^{b}	5.0 ± 3.5^{b}	5.6 ± 3.7^{b}	6.4 ± 1.2^{b}
R^2_{La}	0.977	0.981	0.984	0.984	0.997
p-value	< 0.0001	< 0.0001	< 0.0001	< 0.0001	< 0.0001
$Y_{L/X}$ (g L_a/g X)	0.552 ± 0.153^{a}	0.797 ± 0.116^{a}	0.767 ± 0.126^{a}	0.799 ± 0.138^{a}	0.741 ± 0.101^{a}
$R^2_{La/X}$	0.800	0.989	0.994	0.994	0.994
p-value	< 0.05	< 0.0001	< 0.0001	< 0.0001	< 0.0001
$Y_{L/g}$ (g L_a/g G)	7.37 ± 2.44^{a}	7.78 ± 1.35^{a}	3.65 ± 1.25^{b}	4.14 ± 1.50^{b}	3.28 ± 1.13^{b}
$R^2_{La/g}$	0.772	0.990	0.973	0.967	0.994
p-value	< 0.05	< 0.001	< 0.001	< 0.001	< 0.0001

Table S12. Numerical values and confidence intervals for parameters obtained from mathematical modelling of the experimental data from *P. fluorescens* and *Phaeobacter* sp. (*X* and *Pr*), grown on media summarized in Table S4, by equations [1-6]. R^2 are the determination coefficients among experimental and predicted data. p-value was estimated by F-Fisher test. Different letters in each column indicate a significant difference between media ($p<0.05$). NS: not significant.

		**Pf**		
**Parameters**	**Medium A**	**Medium C**	**Medium D**	**MM**
X_m **(g/L)**	0.38 ± 0.02^{a}	0.60 ± 0.05^{b}	0.39 ± 0.01^{a}	0.97 ± 0.10^{d}
v_x **(g L^{-1}h^{-1})**	0.035 ± 0.013^{a}	0.063 ± 0.033^{a}	0.050 ± 0.007^{a}	0.051 ± 0.019^{a}
λ_x **(h)**	4.1 ± 2.2^{a}	3.1 ± 2.9^{a}	2.4 ± 0.7^{a}	2.4 ± 2.3^{a}
R^2_X	0.986	0.986	0.998	0.974
p-value	< 0.0001	< 0.0001	< 0.0001	< 0.0001
$Y_{X/P}$ **(g X/g Pr)**	0.247 ± 0.053^{a}	0.476 ± 0.114^{b}	0.736 ± 0.251^{b}	1.48 ± 0.29^{c}
m_P **(g X g^{-1} Pr h^{-1})**	0.028 ± 0.012^{a}	0.035 ± 0.009^{a}	0.055 ± 0.028^{a}	0.024 ± 0.010^{a}
$R^2_{X/Pr}$	0.989	0.969	0.946	0.984
p-value	< 0.001	< 0.001	< 0.001	< 0.001
		**Pha**		
X_m **(g/L)**	0.27 ± 0.01^{a}	0.34 ± 0.01^{b}	0.17 ± 0.01^{c}	0.72 ± 0.04^{d}
v_x **(g L^{-1}h^{-1})**	0.017 ± 0.002^{a}	0.027 ± 0.014$^{a.b}$	0.031 ± 0.007^{b}	0.061 ± 0.019^{c}
λ_x **(h)**	7.8 ± 1.2^{a}	3.5 ± 1.9^{b}	3.2 ± 0.7^{b}	4.4 ± 2.1^{b}
R^2_X	0.998	0.994	0.969	0.989
p-value	< 0.0001	< 0.0001	< 0.0001	< 0.0001
$Y_{X/P}$ **(g X/g Pr)**	0.341 ± 0.061^{a}	0.496 ± 0.102$^{a.c}$	0.884 ± 0.199^{b}	0.772 ± 0.207$^{b.c}$
m_P **(g X g^{-1} Pr h^{-1})**	0.048 ± 0.017^{a}	0.048 ± 0.012^{a}	0.083 ± 0.009^{c}	0.010 ± 0.008^{d}
$R^2_{X/Pr}$	0.996	0.984	0.966	0.994
p-value	< 0.0001	< 0.001	< 0.001	< 0.001

Table S13. Summary Table of Mass Yields per kg of Squid Pens Treated. L. plantarum: Lb; L. mesenteroides (Ln)

Process Step	Product	Yield per kg squid pens
Deproteinization	Chitin (dry weight)	600 g
Deacetylation	Chitosan (dry weight)	350 g
Effluent valorization	Lactic acid (Lb)	937 g
Effluent valorization	Lactic acid (Ln)	949 g
Effluent valorization	Biomass (Lb)	147 g
Effluent valorization	Biomass (Ln)	234 g

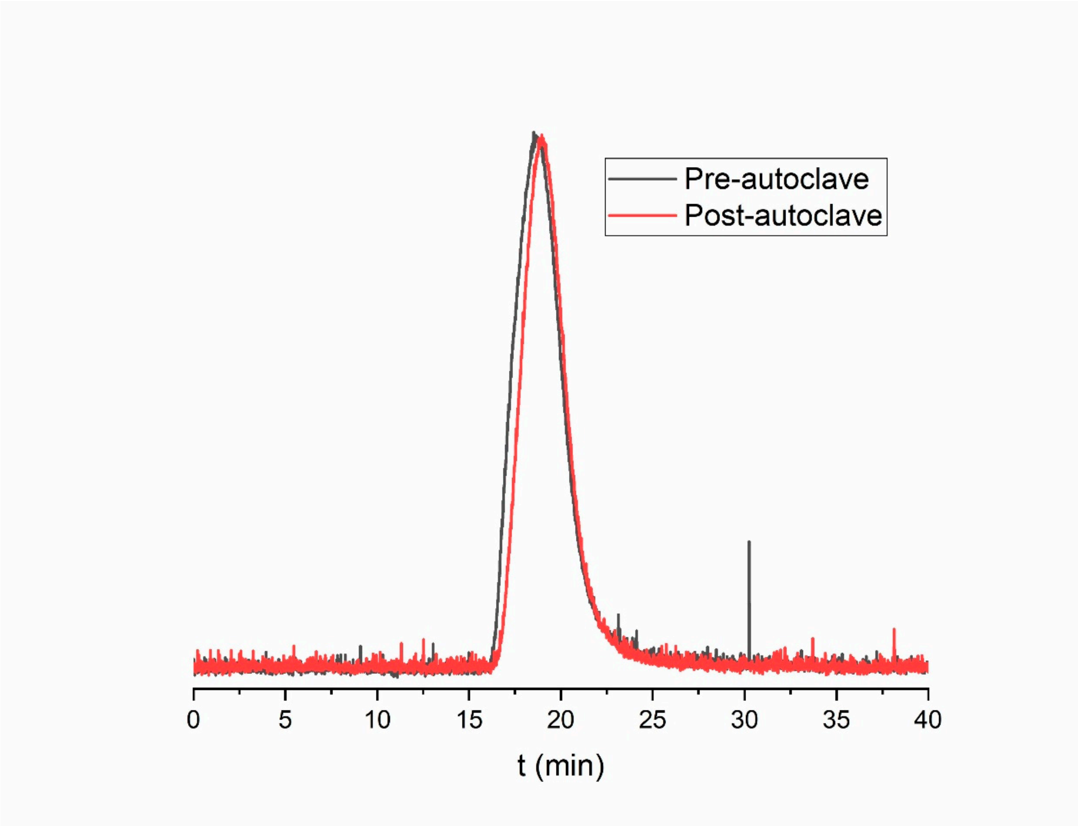

Figure S1. Gel permeation chromatography eluogram (right angle light scattering signal) of pre-autoclave and post-autoclave chitosan (251 kDa). Very close match of peaks evidence a negligible autoclaving (121ºC, 15 min) effect on chitosan molecular weight. Please refer to section *2.5. Polymer characterization* for details about the analytical details.

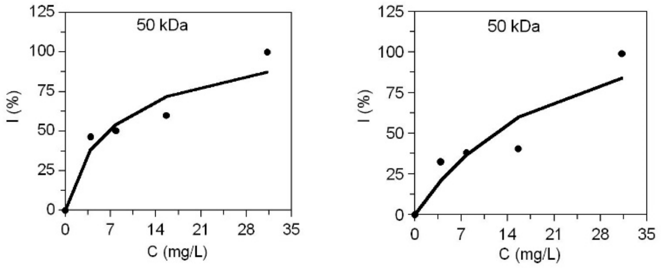

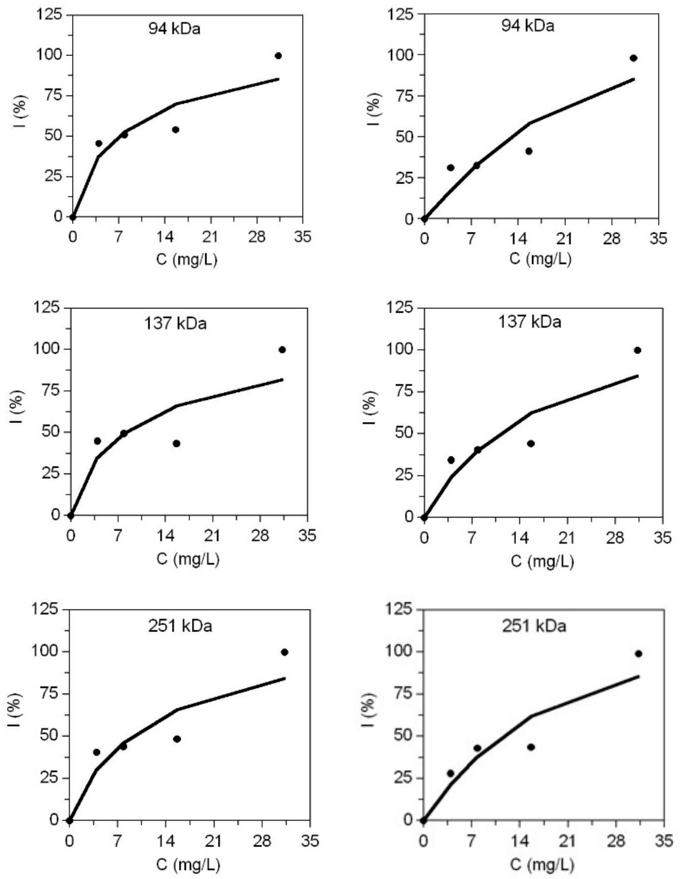

Figure S2. Dose-response relationships between the concentration of chitosan and the growth inhitibition (in terms of OD_m) of *E. coli* (left) and *B. cereus* (right) for each chitosan molecular weights studied. These DR profiles are fitted to the Weibull equation (lines).

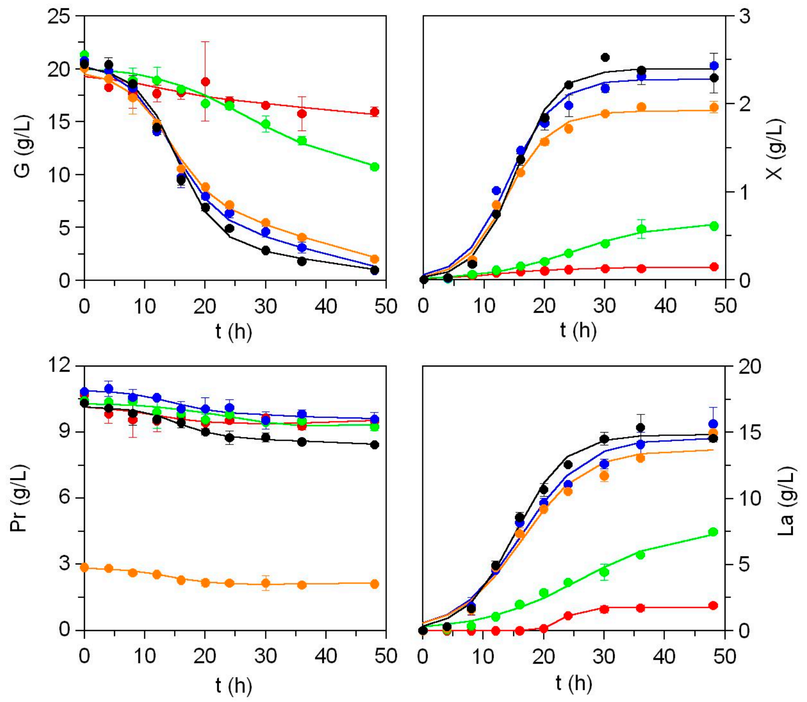

Figure S3. Biomass dynamics, lactic acid productions and nutrients uptakes for Lb growing in the media discribed in Table S3. Experimental data of biomass (*X*), lactic acid (*La*), proteins (*Pr*) and glucose (*G*) were fitted by the equations displayed in Table S5 (continuous lines). Medium A (●), medium B (●), Medium C (●), Medium D (●) and MRS (●). Error bars represent the confidence intervals for n = 3 and α = 0.05.

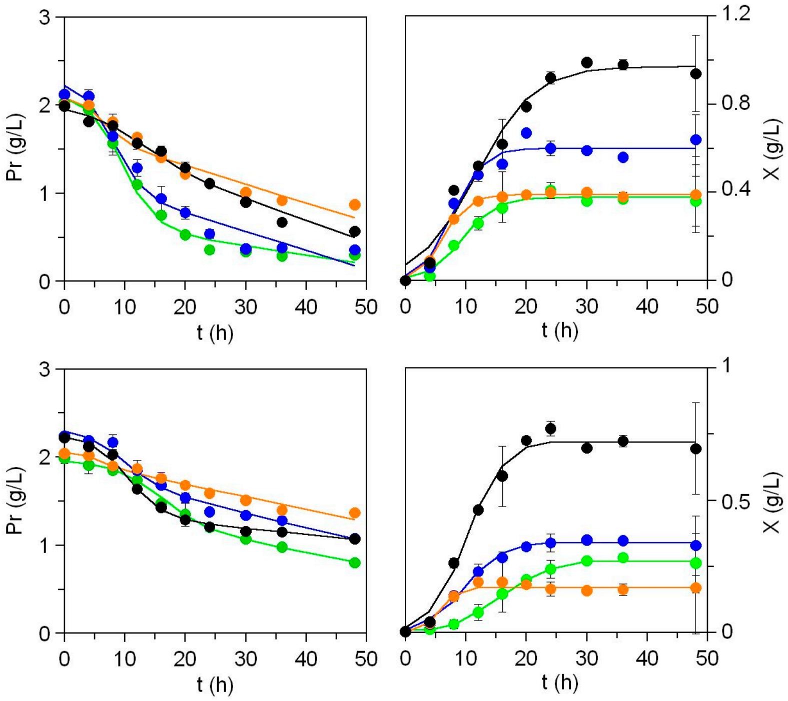

Figure S4. Culture kinetics of Pf (top) and Pha (bottom) in the media discribed in Table S4. Experimental data of biomass (*X*) and proteins (*Pr*) were fitted by the equations displayed in Table S5 (continuous lines). Medium A (●), Medium C (●), Medium D (●) and MM (●). Error bars represent the confidence intervals for n = 3 and α = 0.05.
